# Computational peptide discovery with a genetic programming approach

**DOI:** 10.1007/s10822-024-00558-0

**Published:** 2024-04-03

**Authors:** Nicolas Scalzitti, Iliya Miralavy, David E. Korenchan, Christian T. Farrar, Assaf A. Gilad, Wolfgang Banzhaf

**Affiliations:** 1 BEACON Center of Evolution in Action, Michigan State University, East Lansing, MI USA; 2 Department of Computer Science and Engineering, Michigan State University, East Lansing, MI USA; 3 Athinoula A. Martinos Center for Biomedical Imaging, Department of Radiology, Massachusetts General Hospital and Harvard Medical School, Boston, MA USA; 4 Department of Chemical Engineering, Michigan State University, East Lansing, MI USA; 5 Department of Radiology, Michigan State University, East Lansing, MI USA

**Keywords:** Peptide discovery, Genetic programming, CEST MRI, Contrast agent, Regular expressions, Evolutionary algorithm

## Abstract

The development of peptides for therapeutic targets or biomarkers for disease diagnosis is a challenging task in protein engineering. Current approaches are tedious, often time-consuming and require complex laboratory data due to the vast search spaces that need to be considered. *In silico* methods can accelerate research and substantially reduce costs. Evolutionary algorithms are a promising approach for exploring large search spaces and can facilitate the discovery of new peptides. This study presents the development and use of a new variant of the genetic-programming-based POET algorithm, called POET_*Regex*_, where individuals are represented by a list of regular expressions. This algorithm was trained on a small curated dataset and employed to generate new peptides improving the sensitivity of peptides in magnetic resonance imaging with chemical exchange saturation transfer (CEST). The resulting model achieves a performance gain of 20% over the initial POET models and is able to predict a candidate peptide with a 58% performance increase compared to the gold-standard peptide. By combining the power of genetic programming with the flexibility of regular expressions, new peptide targets were identified that improve the sensitivity of detection by CEST. This approach provides a promising research direction for the efficient identification of peptides with therapeutic or diagnostic potential.

**Supplementary Information:**

The online version contains supplementary material available at 10.1007/s10822-024-00558-0.

## Introduction

### Peptide-based therapies and diagnostics

Peptides are molecules composed of amino acids (AA) joined by peptide bonds. They are short sequences usually comprised of 2 to 50 AAs. Peptides are one of the cornerstones of living organisms and participate in many metabolic and physiologic activities, acting as hormones (e.g. insulin) [1], neurotransmitters [2], antimicrobial agents [3] or venoms [4–6]. Because of their intrinsic physicochemical properties (e.g. high selectivity and efficacy, low toxicity), peptides are a powerful target for therapeutic development [7–10]. Indeed, since the first use of insulin over 100 years ago, peptides have been extensively studied as potential targets for various therapeutic applications such as cancer [11–13] or diabetes treatments [14]. They are also used to cater to a wide range of chronic [15] or rare diseases [16, 17], and have the potential to be used as vaccines [18]. More recently, they have been used to fight against Covid-19 [19]. In addition, peptides can serve as biomarkers for disease diagnostics. Indeed, human fluids, such as blood plasma, contain a wide range of proteins and peptides that represent a large source of physiologic information. Peptide biomarkers are used in different disease diagnostics such as cancer [20, 21], type II diabetes [22] or neurodegenerative disorders such as Alzheimer’s disease [23]. Peptides are also used in imaging diagnostics such as positron emission tomography [24], single-photon emission computerized tomography [25] and chemical exchange saturation transfer (CEST) magnetic resonance imaging (MRI) [26–28].

### Protein engineering

Because of their wide range of applications in therapies and diagnostics and their advantages over traditional drugs, peptides have tremendous potential in biomedical fields. However, despite billions of years of evolution, the protein and peptide search space is not fully explored. Thus, the discovery and design of new peptides is a gargantuan task that researchers are trying to solve through two main approaches: (i) rational design and (ii) Directed Evolution (DE). In rational design, scientists use knowledge of a protein/peptide (e.g. crystalline structure) to optimize a new valuable target with desired functional and structural properties [9, 29]. DE is based on a model protein with similar function to the desired one, however, does not require more prior knowledge. This approach uses iterative mutagenesis and screening, which are the main operators to generate new targets guided by artificial evolution [30–32]. Unfortunately, these methods are not the Holy Grail for generating new therapeutic/diagnostic peptides, and some disadvantages slow down the research [33]. Indeed, these methods are time-consuming and costly. Moreover, necessary prior knowledge and wet lab experiments can pose limits. Finally, the search space is extremely complex, and the optimization trajectories could easily get stuck in local optima rather than global.

### Computer-aided design of peptides

To overcome these problems, researchers have started to use new computational methods generally based on machine learning (ML) and optimization techniques. The advent of artificial intelligence (AI) has allowed the development of new methods and tools to predict the structure or the function of proteins and peptides [34, 35]. Furthermore, Evolutionary Algorithms (EAs) are widely used in the computational design of proteins and peptides [36, 37]. EAs are bioinspired metaheuristic optimization algorithms and are powerful tools to solve search and optimization problems [38]. One of the main advantages of EAs is their ability to explore a large search space [39]. Considering the creation of a peptide with 12 AAs (using only 20 classic AAs), the search space has already ${20^{12}}$ possible targets. Thus, EAs should be very suitable for navigating in this space in order to discover new therapeutic/diagnostic peptides.

### Related work

The journey of EAs in protein design is relatively recent, with about 30 years of research. Many works focus on the prediction of the three-dimensional structure of proteins or their function, or on motif discovery. In the 1990 s Unger et al. developed an approach based on a genetic algorithm (GA) for protein folding simulations [40]. In 1995, Koza et al. exploited genetic programming (GP) to evolve motifs for the identification of the D-E-A-D box family of proteins and for the detection of the manganese superoxide dismutase family [41]. One year later, Yokobayashi et al. developed a method based on DE and a GA to generate new peptides with more efficient inhibitory activities. By carrying out artificial evolution, they obtained an improvement of more than 90% for some peptides [42]. Hu et al. proposed a GP method to identify patterns in protein sequences. They used a PROSITE [43] pattern representation, close to regular expressions (REs) for representing individuals [44]. Based on Hu’s works, Ross et al. used stochastic REs as a new representation language for protein sequences classification. A GP algorithm is then applied to evolve the stochastic REs and obtained promising results [45]. Heddad et al. also used a GP algorithm to generate and evolve RE-based classifiers. Their approach uses these classifiers to determine the nuclear localization of a protein [46]. In 2005, Seehuus et al. exploited a GP algorithm to discover patterns in biological sequences. They applied linear GP to evolve individuals represented by REs. Their method has shown comparable results to those found in PROSITE [47]. In 2007, Yagi et al. proposed a new approach called ’*in silico* panning’ for the selection of peptide inhibitors. They exploited a docking simulation associated with a GA to evolve target peptides. Interestingly, they showed the effectiveness of *in silico* evolution combined with experimental data [48]. In 2011, Becerra et al. proposed a procedure to predict the three-dimensional structure of proteins. Their strategy is based on a multi-objective parallel *ab initio* algorithm. They used the NSGA-II multi-objective GA to optimize the energetic contributions of the protein [49]. Yousef et al. combined a GA and protein free energy minimization calculations for the prediction of the three-dimensional structure of proteins [50]. Recently, Yoshida et al. used a combination of a GA and an *in vitro* evaluation. The individuals are potential antimicrobial peptides, and the fitness function is the wet lab test. With this *in silico*-*in vitro* approach, they obtained promising results and identified 44 new antimicrobial peptides with 160-fold efficiency [51]. In the same year, Porto et al. published an approach based on a GA to design a guava antimicrobial peptide (one of the first plant-based peptides) [52].

### Development of the POET_*Regex*_ tool

in this context, we developed a new computational approach based on GP for new peptide discovery, called POET_*Regex*_. Our method is an extension of the initial version of the Protein Optimization Engineering Tool (POET) [53, 54]. This extension replaces the motif discovery mechanisms of POET with a more comprehensive process by incorporating regular expressions (REs). We have modified the representation of individuals by evolving lists of REs instead of lists of motifs of contiguous AAs, to identify relevant patterns with more flexibility. The specific characteristics of the elements (operators) comprising the syntax of REs enable them to effectively identify motifs through the combination of these elements. The second enhancement to POET involves the weight adjustment step, also called the training step. Unlike the initial version of POET, where weights are randomly assigned, here, the weights of an RE are adjusted based on the significance of that motif (see Materials and Methods). Therefore, the main objectives of this study are to evolve protein-function models based on REs using a GP algorithm, to obtain a trained model, which can then be used to generate new peptides for a specific problem. Evolving REs with a GP algorithm is a method capable of exploring a huge search space and finding good solutions. REs are powerful tools and are widely used in computational evolutionary research for pattern or motif discovery, and text extraction [55–63].

As proof of concept, we apply our method to address the problem of the sensitivity of peptides to be detected by MRI with chemical exchange saturation transfer. CEST is an MRI contrast approach where exchangeable protons are saturated by radiofrequency irradiation [64]. This saturation is then transferred to water protons and the signal can be detected. Contrast detection by CEST has great potential for clinical imaging [65]. Initially, poly-L-lysine (composed of 12 lysine residues) was used as a CEST contrast agent to pave the way for the search for new sensitive agents and is now considered the gold standard [66]. Since peptides are interesting agents for CEST contrast [27], we used our method to train a model based on GP with CEST data, and we discovered new peptides that provide high CEST contrast.

## Materials and methods

This section describes the data used, the GP algorithm combined with REs and the different steps to obtain predicted peptides and validate them experimentally.

### Datasets

Having good quality and curated data is a fundamental requirement to train an accurate model. Unfortunately, high-quality data is rare, and databases often contain a significant amount of erroneous data [67]. Therefore, the curated dataset used in this study is mainly based on data from nuclear magnetic resonance (NMR) measurements of CEST contrast from various peptide samples dissolved in a buffer solution [54, 55]. The dataset contains 158 sequences of peptides ranging from 10 to 13 AAs in length. The 20 standard AAs were used and the CEST values were measured at 3.6 ppm, corresponding to amide proton exchange. Then, two sub-datasets are generated, one to train the models (training set) and the other to evaluate model performances on unseen data (test set). The training set contains 127 ( 80%) randomly drawn sequences, and the test set contains the remaining 31 ( 20%) sequences. The whole dataset is available as Additional file 1: Table S1.

### Motif database construction

To train the model by adjusting the weight of each RE, the algorithm uses a list of motifs extracted from data in the training set. Extracted motifs are the basic units of information in the evolutionary process. These motifs are recovered using a sliding window of a size varying from 2 to 6 AAs, which is applied to each sequence (single AAs are also extracted). To determine whether a motif should be favored or not, it is assigned a class based on the CEST value of the sequence from which the motif was extracted. Class 0 is chosen if the motif has a negative impact on the peptide results (< threshold) and class 1 if the motif has a positive impact on the peptide results (> threshold). The threshold is defined based on the experimental target and was set in this study to a value of 12.5, which corresponds to the CEST value of the poly-L-lysine peptide (K12) in the dataset, the ”gold-standard peptide” [64, 68]. Since each training sequence is associated with a CEST value, it is possible to associate a CEST value with each extracted motif. However, the same motif may be present in several training sequences with different CEST values. To address this issue, a strategy called ‘occurrence’ is implemented to associate a CEST value with a motif. To do this, the number of motifs present in both class 1 and class 0 sequences is counted. The class exhibiting the highest number of motifs is chosen, and the average value is calculated. The final motif database (MDB) contains 5,360 motifs from 2 to 6 AAs, each associated with a CEST value and a class.

### Sequence identity

In order to verify that there is no over-representation of sequences with identical motifs or identical sequences in the dataset, a sequence similarity search was performed on all sequences in the dataset to calculate the percent identity per pair. This calculation was done according to the following formula:

$$\% {\text{Identity}}=\left( {\frac{{\# \;{\text{of}}\;{\text{Identical}}\;{\text{AA}}}}{{{\text{Sequence}}\;{\text{Length}}}}} \right) \times {{100}}$$

### Regular expressions

A RE is a sequence of characters, including operators and variables, that describes a search pattern in a target text according to a precise syntax. The operators used for this study are presented in Table 1. REs are implemented with the *re* library of Python version 2.2.1.

**Table 1:** RE operators used in this study

Operator	Symbol	Description	Arity
Concatenation	$\emptyset$(invisible)	Concatenate two elements	2
Alternative choice (or)	|	Choice between two elements	2
Quantifier 1,n	+	Define a group present once or *n* time	1
Curly braces	{ }	Define the number of times the element is repeated	1
Bracket	[ ]	Define a list of choice between elements in the bracket	1
Excluding bracket	[⌃]	Define a list of choice between elements that are not in the bracket	1
Parenthesis	( )	Define a group	1

### Model evolution using genetic programming

GP algorithms [69, 70] are powerful evolutionary computing techniques, a branch of AI widely used in different fields such as engineering or bioinformatics [71]. GP is a stochastic algorithm (an extension of the Genetic Algorithm) inspired by the concepts of Darwinian evolution and is useful for automatically solving complex optimization problems [69].

**Fig. 1:**
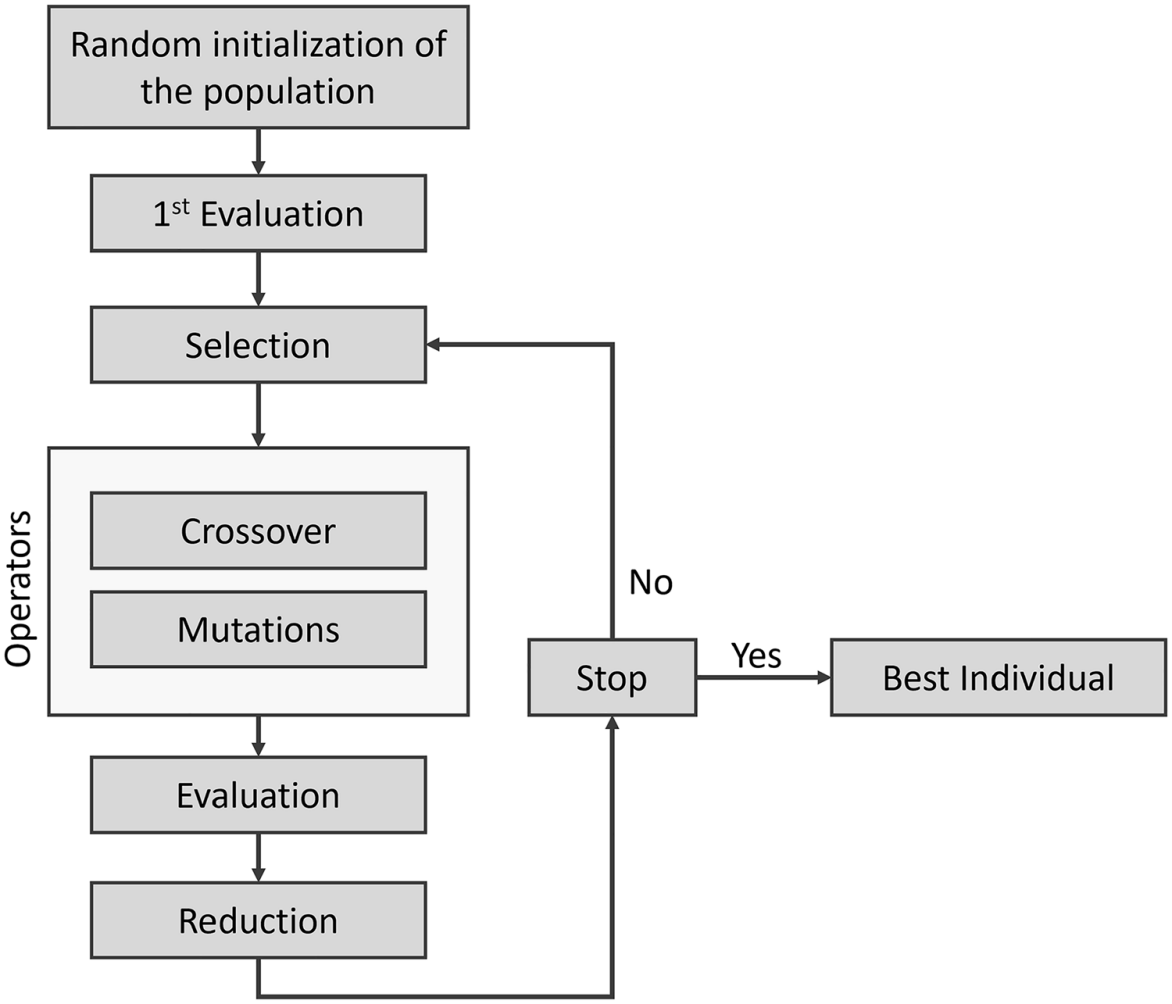
Classical evolutionary cycle of a GP algorithm

This type of algorithm is designed to explore a large search space and generate potential solutions through evolutionary mechanisms: selection, recombination (or crossover) and mutation. The solutions represent individuals in the population *P* that GP will evolve. In each iteration, all individuals are evaluated based on a fitness function (here: Pearson correlation coefficient) to obtain a fitness value, which is used to rank each individual according to their ability to solve the problem (here the ability to generate new peptides with the highest CEST contrast at 3.6 ppm). Two evolutionary operators are then typically applied: crossover and mutation. During crossover, two individuals (”parents”) are selected and a part of parent 1 is exchanged with a part of parent 2. This operation generates two new individuals (”offspring”) with a mix of the characteristics of their parents. Consequently, the size of population *P* increases. The mutation operators are then applied. Depending on the problem and the representation of the individual, these operators can vary. Typically, mutation operators involve the addition, deletion or substitution of an element of the target individual. Finally, the new individuals are evaluated using the fitness function and in the reduction step only the *S* best individuals (with *S* the initial size of the population *P*) are selected to be included in the population in the new generation to continue their evolutionary journey in the run. The evolutionary cycle ceases when a stop condition is reached, such as time, number of runs, or the algorithm finding a satisfying solution. At the end of the evolutionary process, we obtain the best individual representing the best evolved model, which we use for peptide generation. Figure 1 illustrates the evolutionary cycle of GP used in this study.

### Representation of individuals

Unlike in a GA, in which individuals are fixed-length strings, individuals in GP are represented by computational programs, usually as a tree structure (an acyclic network consisting of nodes connected by edges) [70] or linear sequences (such as instructions) [72]. GP manipulates these programs with different operations, however the tree structure also allows the use of the syntax of REs.

**Fig. 2:**
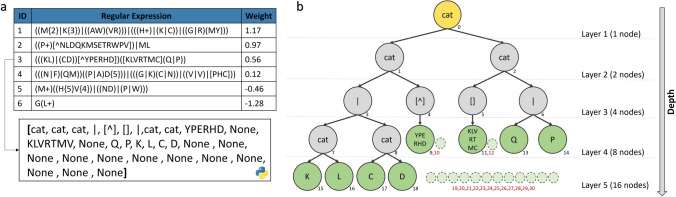
**a** Representation of an individual (a protein-function model) as a list of rules with 3 columns (ID, regular expression pattern and weight). An example (RE3) is represented as a built-in list structure in Python, where a parent node *i* has 2 children: (i*2)+1 and (i*2)+2. **b** Representation of RE3 as a binary tree. The yellow node is the root, grey nodes are the internal nodes and green nodes are the leaves. The small dotted nodes with red numbers are unexpressed nodes represented by “None”

In our method, an individual is a protein-function model represented by a list of rules, with each rule being composed of a number (ID), a regular expression, and a weight (Fig. 2a). Each RE is represented by a binary tree implemented as a list where node *i* is the parent, and node (i*2)+1 and (i*2)+2 are children (Fig. 2b). Each internal node represents an operator, and each leaf (or terminal node) represents a variable. The maximal depth of a tree is 6, which prevents having REs that are too long and time-consuming to evaluate. REs are randomly generated using the ramped half-and-half strategy [73] to create a population with heterogeneous individuals. Initially, individuals have a list of between 1 and 8 REs with weights of 0.

### Evolutionary operators

Three main evolutionary operators are used in this study: (i) selection, (ii) crossover, and (iii) mutation. The next paragraphs describe these operators in more detail.

#### (i) Selection

The selection operator plays a key role in evolution by determining which individuals will proceed to the next steps of evolution (crossover and mutations). The individuals selected for crossover are referred to as parents. One commonly used selection method in GP is tournament selection [74]. In tournament selection, a random sample of *k* individuals (which represents the size of the tournament) is chosen from the population with replacement. The best individual in the tournament (i.e., the one exhibiting the greatest fitness value) is then selected to become a parent. Tournament size influences the selection pressure: a higher value of *k* reduces the likelihood of selecting a bad individual, thus increasing selection pressure, while a lower value of *k* increases the chance of selecting a bad individual, thus lowering selection pressure. In this study, a tournament size of *k*=5 has been chosen.

#### (ii) Crossover

**Fig. 3:**
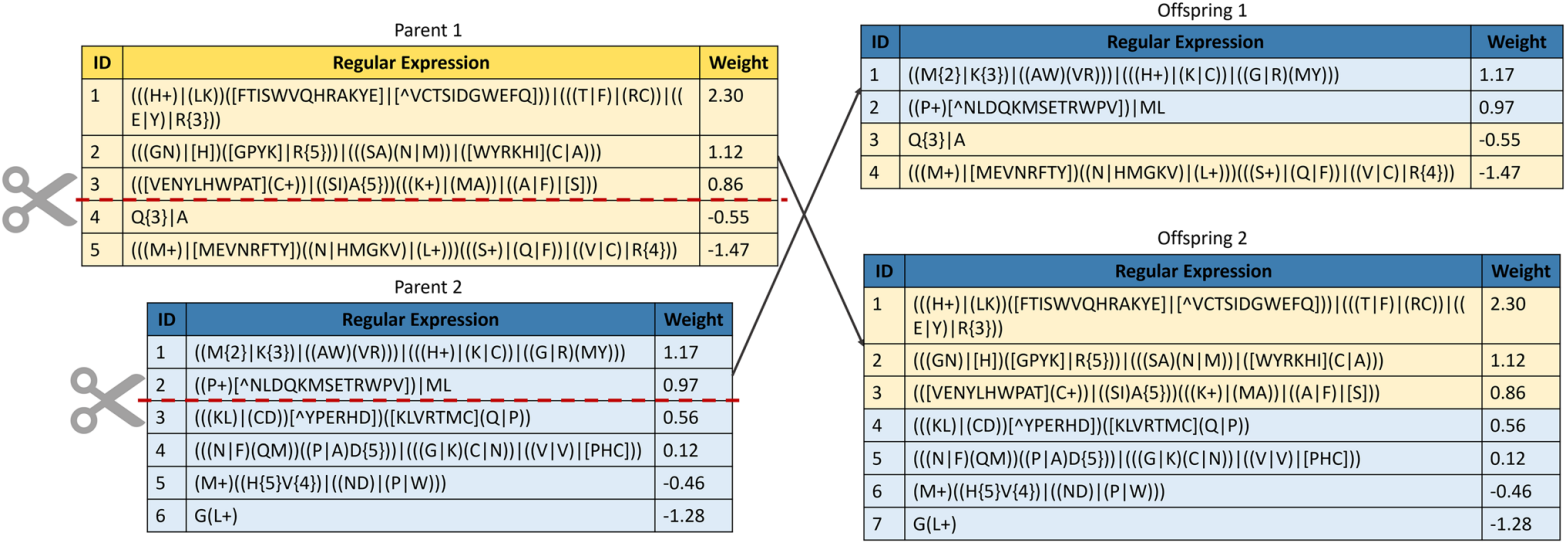
Representation of the one-point crossover: a part of parent 1 is merged with a part of parent 2 to produce offspring

The crossover operator involves combining a parts of both parents to generate offspring. A one-point strategy is used, wherein a point in a parent is selected cutting it to form two parts (A and B). This process is repeated with the second parent. The next step involves exchanging parts between parents to create two offspring with a mix of elements from both parents. The crossover operator provides diversity and can preserve important features, which make it a widely used method for generating offspring with desirable traits in evolutionary algorithms. Figure 3 illustrates the one-point crossover operator used in this study. However, the disadvantage of crossover is that it may converge to a local maximum during the evolutionary process [75] because no new elements are introduced into the population.

#### (iii) Mutations

Mutations allow the exploration of the search space by inducing new elements into an individual increasing the diversity of the population. This study implements two groups of mutations, with the first group targeting the individual as a whole and the second group targeting specific REs. Each individual or RE has a mutation rate of 10%.

**Fig. 4:**
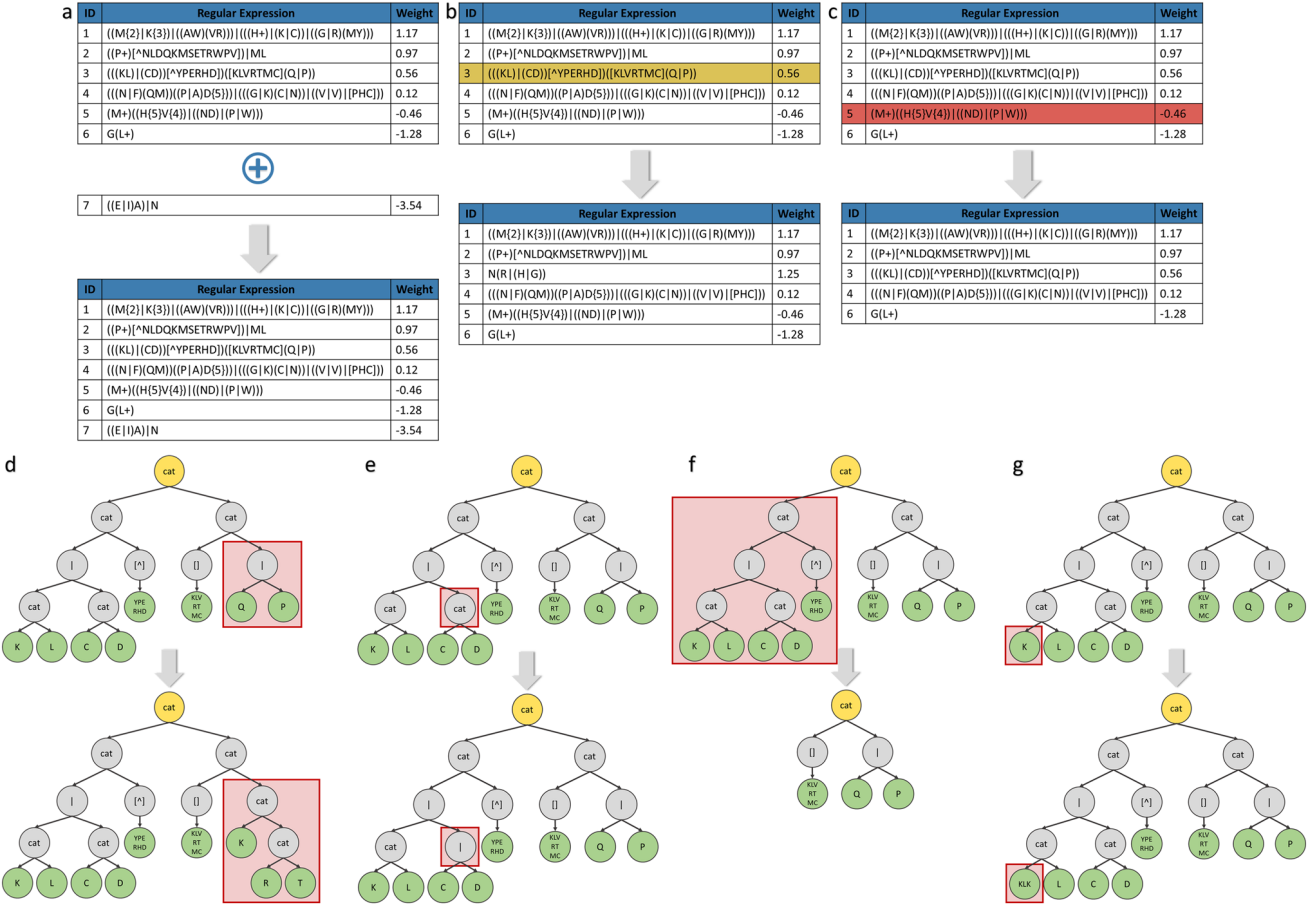
Representation of each type of mutation. **a** Addition of a new rule in the list of rules. **b** Replacement of a rule by a new rule. **c** Deletion of an existing rule in the list of rules. **d** Replacement of a branch of the tree. **e** Exchange of a node. **f** Deletion of a subtree. **g** Add one or more AAs to a leaf

Group I contains three types of mutations:

- Addition of a new rule (Fig. 4a): If the number of rules of an individual does not exceed a maximum of the number of rules allowed, then a new rule is randomly generated and added to the list of rules of the individual.
- Replacement of an existing rule (Fig. 4b): An existing rule is randomly selected and replaced by a newly generated rule.
- Deletion of an existing rule (Fig. 4c): If the list of rules of an individual has at least two or more rules, then an existing rule is randomly selected and removed from the rule list.

Group II contains 4 types of mutations, only impacting one RE:

- Replacement of a branch of the tree (Fig. 4d): A branch of the tree (subtree) is randomly selected and replaceed with a new randomly generated one (its depth can vary).
- Exchange of a node (Fig. 4e): A node is randomly selected and its value is changed. For example, cat ($\varnothing$) becomes or (|), including bracket ([ ]) becomes excluding bracket ([⌃ ]). If the node contains an AA, it is replaced by another random AA, and if the node is the value contained in the curly braces, then the value is replaced by another one.
- Deletion of a subtree (Fig. 4f): A subtree is randomly selected and deleted.
- Addition of new AA in a leaf (Fig. 4g): 1 to 4 new random AAs are added in the leaf, to create a specific motif.

Figure 4 illustrates the different mutation operators used in this study.

### Evaluation with the fitness function

Each individual is assigned a fitness value during the evaluation step. This fitness value reflects the degree of adaptation to the problem, with higher fitness values indicating better adaptation. In this study, a Pearson correlation coefficient-based objective function was used to evaluate the models, which attempts to obtain the best correlation between a predicted score and a true CEST value for each peptide sequence (details are given in section ’Determining the Fitness Value’ below). This coefficient ranges from -1 (indicating a strong negative linear correlation) to 1 (representing a strong positive linear correlation), while a value of 0 signifies no correlation. To compute the fitness value of an individual, each RE of its rule list is (i) trained (i.e., its weight is adjusted) and (ii) the fitness value is determined. These steps are parallelized on 20 CPU cores to expedite execution.

#### (i) Weight adjustment during the training step

As mentioned earlier, each RE is associated with a weight determining its importance in the model. The weights of the REs are initialized to 0 at the beginning of the algorithm. In each generation, fitness values are computed to determine the best individuals. The first step consists in adjusting the weight of each RE based on the motif data base (MDB) constructed from the training dataset. Each RE is tested against each training sequence, and the resulting matches (motifs) are extracted. For instance, if the RE ‘*[PNYIQ]K+*’ is applied to the sequence APVPKKPRLL, it will identify the motif ’PKK’. This motif is associated with a CEST value in the MDB. The score of the RE on this sequence corresponds to the CEST value multiplied by the size of the motif. If the CEST value is equal or greater than the threshold (here 12.5), then we add the value to the final score; otherwise, we subtract it. The final weight of the RE is the sum of all scores obtained on each training sequence, as shown in the following equation:

$$\begin{aligned} \text {{Final\_Weight}} = \left( \sum _{i=1}^{n} \left\{ \begin{array}{ll} +CEST_{\text {{motif}}_{\text{i}}} \times Size_{\text {{motif}}_{\text{i}}}, &{} \text {{if CEST}} \ge T  \\  -CEST_{\text {{motif}}_{\text{i}}} \times Size_{\text {{motif}}_{\text{i}}}, &{} \text {{if CEST}} < T \end{array} \right. \right) \end{aligned}$$

with *n* the number of extracted motifs in the training sequences and *T* the threshold.

#### (ii) Determining the fitness value

After the training step, each RE of an individual is applied to each sequence in the training set to ensure that the model effectively generalizes the data. The *predicted_score* is the sum of all final weights of the REs that match in the sequence. This *predicted_score* is then combined with the true CEST value of the sequence evaluated by the individual, and the Pearson correlation coefficient, corresponding to the objective function, is used to calculate the strength of the linear relationship between the *True_CEST* and the *predicted_score*, as shown in the following equation:

$$\begin{aligned} {r = \frac{ \sum _{i=1}^{n}(PS_i -\overline{PS})(CEST_i-\overline{CEST}) }{ \sqrt{\sum _{i=1}^{n}(PS_i -\overline{PS})^2}\sqrt{\sum _{i=1}^{n}(CEST_i-\overline{CEST})^2}},} \end{aligned}$$

with *r* the Pearson correlation coefficient, *PS* the predicted score, $\overline{PS}$ the mean of the predicted scores, *CEST* the true CEST value and $\overline{CEST}$ the mean of the CEST values. The closer the fitness value (*r*) is to 1, the better the individual (performance of the model). This means that the predicted scores for the sequences are closely related to their true CEST values. In other words, using the model, predictions can be made about CEST values, and as the predicted score increases, the associated CEST value is also expected to increase, reflecting a positive correlation between the two.

To prevent overfitting, cross-validation is performed using the k-fold method with k=6. Consequently, the fitness value of an individual corresponds to the average performance of the (k-1)-folds used with the training data, while the average of the remaining 1-fold are used to evaluate the algorithm’s behavior and identify any signs of overfitting.

### Elitism

Before each mutation and crossover step, the best individual (elite) is extracted and automatically included in the next generation with no change, to prevent the algorithm loosing the current best solution.

### Peptide prediction with the best evolved model

Once the algorithm has reached a stopping condition, such as the maximum number of generations reached or the fitness value plateauing, indicating that the algorithm has reached a local optimum, the best evolved individual can be used as a model for generating new peptides. Therefore, a higher predicted score should imply a higher CEST value for the predicted peptide.

*In silico* DE coupled with the best evolved POET_*Regex*_ model is employed for the prediction of new peptides. This approach has already been successfully applied in previous studies [42, 53, 76]. Three DE experiments were conducted with different cycle numbers (10, 100, and 1000 cycles). Insufficient cycles could result in heterogeneous peptides and hinder convergence of the algorithm, while a high number of cycles may lead to converged results and homogeneous peptides. A library of 1000 peptides is generated randomly at the beginning of each experiment, and the peptide sequences then undergo three steps: mutagenesis, evaluation, and selection. The mutagenesis step consists of introducing random mutations (substitution of an AA) to generate new variants with increased fitness. The evaluation step employs the best evolved/trained model, replacing the long, tedious, and often expensive wet-lab screening process. Each peptide is evaluated using the best POET_*Regex*_ model, which provides a score correlated to the presumed CEST value. If the fitness of the mutated peptide exceeds that of the initial peptide, the mutated peptide will subsequently replace the initial peptide and be selected for the next cycle. In the context of identifying peptides with high CEST contrast, a filter is implemented to exclusively select hydrophilic peptides at the end of the evolutionary process. This filter calculates the sum of the hydrophobicity values of each AA in the peptide (from [77], Additional file 1: Table S2). If the sum is greater than zero, the peptide is selected. Conversely, if the sum is equal to or less than zero, the peptide is eliminated from consideration and classified as non-soluble. Finally, from the remaining peptides, the top 20 are extracted, as they are considered to have the highest potential CEST value among the selected hydrophilic peptides.

### Peptide synthesis and preparation

Each peptide generated by the best POET_*Regex*_ model was synthesized by Genscript USA Inc. (Piscataway, NJ). Peptides were prepared by dissolving 4–5 mg of peptide in 600 $\mu$L of PBS, then titrating the solution to pH 7.25$-$7.30 (measured using a pH electrode calibrated between pH 7 and 10 at room temperature) using 0.1 M HCl or 0.1 M NaOH. Each solution was then pipetted into a separate 5 mm NMR tube.

### CEST NMR measurements

The CEST data were acquired on a 14.1 T vertical-bore Bruker Avance III HD NMR spectrometer with the sample temperature set to 37°C. For each sample, the probe was tuned and matched as soon as the sample temperature was reached and stable, then the sample was shimmed manually on the water proton resonance, and the 90° pulse length was calibrated by finding the 360° zero-crossing and dividing by four. The spin–lattice relaxation time constant (T_*1*_) was measured for each sample using an inversion-recovery sequence modified to include a z-gradient pulse at 5% of the maximum amplitude between the inversion and excitation pulses, to reduce radiation damping [78]. Z-spectra were obtained at least 40 min after the sample temperature probe reached stability, so that the sample had sufficient time to equilibrate. The CEST sequence was an ultrafast z-spectroscopy sequence [79] with the following parameters: 2048 acquired FID points, 42.6 kHz bandwidth, 32 scans, 10 s recovery delay between scans, 5% gradient applied during saturation and acquisition, pulse offset frequency set to be $\sim$3250 Hz higher than the water frequency, 5 s saturation pulse, saturation power varying from $\sim$1.2–5.2 $\mu$T with 10 powers measured per sample. Four dummy scans were performed between each saturation power value. Each sample also included a reference scan (S_*0*_, saturation power = 0 $\mu$T) at the beginning and end of the z-spectroscopy; all samples showed little to no change between the two reference scans, indicating sample stability.

All z-spectral data were processed using custom-written MATLAB scripts, including scripts developed by the research group of Dr. Moritz Zaiss, publicly available on GitHub at

https://github.com/cest-sources. Raw FID data were loaded into MATLAB, zero-filled by a factor of 16, Fourier transformed, and normalized by the first reference scan (saturation power = 0 $\mu$T) to obtain z-spectra. The magnetization transfer ratio asymmetry (MTR_*asym*_) was calculated using the z-spectral amplitudes at ±3.6 ppm and the following equation:

$$\begin{aligned} MTR_{asym} = \tfrac{S(-3.6ppm)-S(3.6ppm)}{S_0} \end{aligned}$$

### Configuration

The computational experiments were performed on Michigan State University’s High Performance Computing Center computers. Each experiment utilized 20 CPU cores and 15 GB of RAM. A configuration file was employed to specify different hyper-parameters of the algorithm, which are summarized in Table 2. The scripts are implemented in python v3.10.6 and are available at the following link: https://gitlab.com/NicolasScalzitti/poet_regex.

**Table 2:** Hyper-parameters used in POET_*Regex*_ experiments

Hyper-parameter	Value
Population size	1000
Number of runs	300
Max RE	30
Crossover probability	0.9
Mutations probability	0.1
Tree depth	6

## Results

### Data

**Fig. 5:**
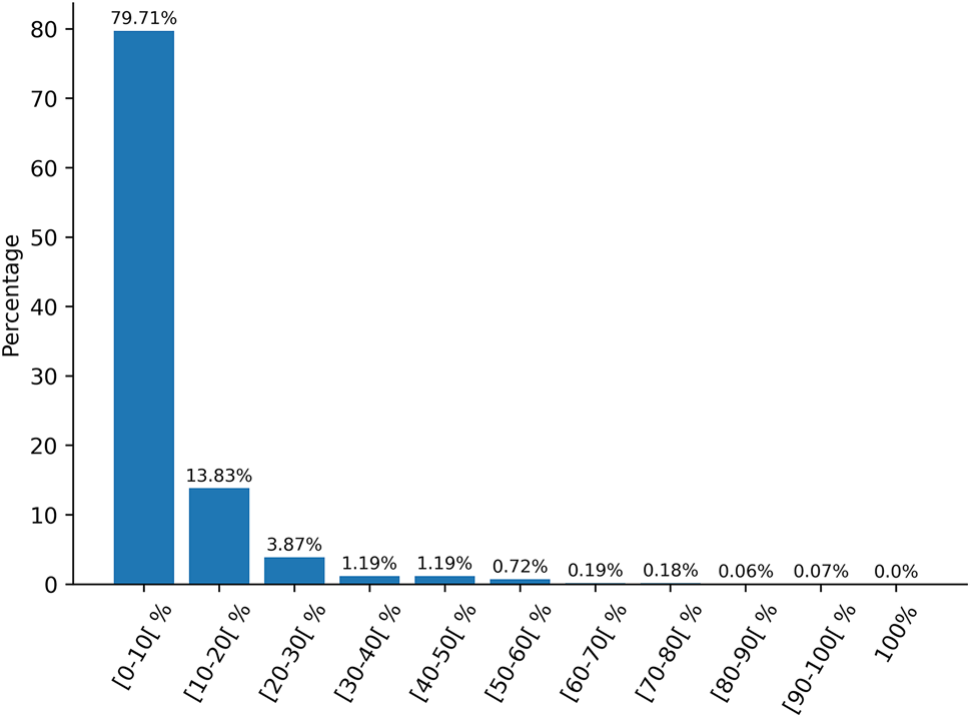
Average pairwise sequence identity in the dataset in percent, with [i–j] indicating values from i (included) to j (excluded)

In order to verify that the dataset contains unique data and that certain sequences are not over-represented, we performed a pairwise sequence similarity calculation on the entire dataset (Fig. 5). The results were averaged and bins of ten percent, showing that most sequences ($\sim$80%) share less than 10% identity, demonstrating that the dataset used is heterogeneous. Only a very small portion of the data (1.22%) have more than 50% identity. Moreover, there are no completely identical sequences.

**Fig. 6:**
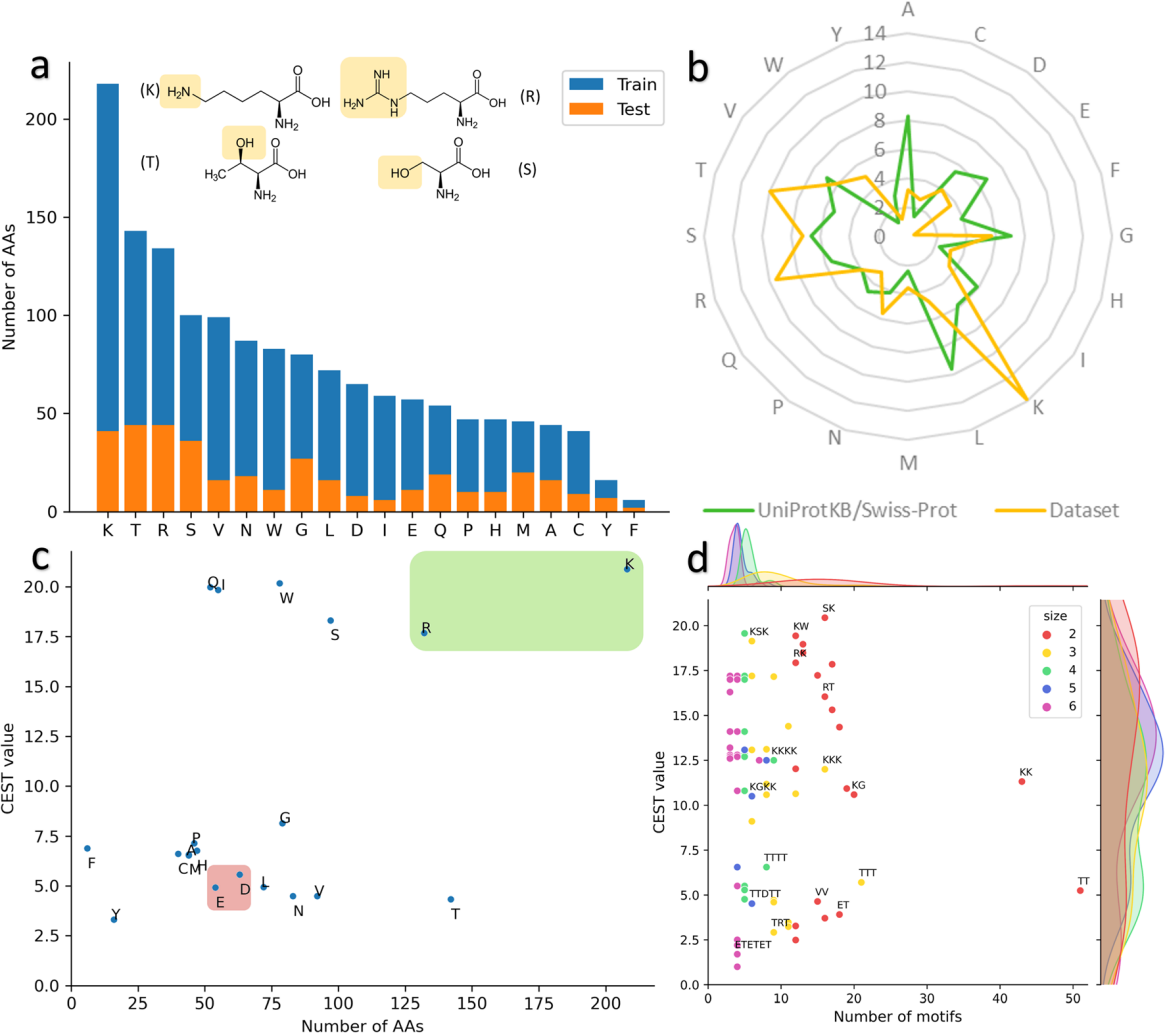
**a** Frequency of occurrence of each AA in both training (blue) and test (orange) sets. Molecules are illustrated for the four most prevalent AAs in the training set, and hydroxyl or amine groups are highlighted. **b** Comparison of the frequency of each AA in our dataset (yellow) and in the UniProtKB/Swiss-Prot database (green). The different values represent the percentage of occurrence. **c** Potential CEST value associated with each AA by occurrence method. The green box represents positively charged AAs, and the red box represents negatively charged AAs. d) Frequency of the 20 most observed motifs (size 2 to 6) in the training set with the associated CEST value

We then conducted a more detailed analysis of the dataset. Initially, we examined the frequency of occurrence of each amino acid (AA) in both the training and test sets, as illustrated in Fig. 6a. Our observations indicate that lysine (K), threonine (T), arginine (R), and serine (S) are among the most commonly occurring AAs in both sets. These AAs are polar and possess either hydroxyl, amine, or guanidine (3 amines) groups. In addition, K and R are positively charged, enabling them to accept protons and be soluble in water. Tyrosine (Y) and phenylalanine (F) are the least frequent AAs in the dataset. These AAs are relatively uncommon in natural proteins, accounting for only 2.92% and 3.86%, respectively. Their hydrophobic and aromatic nature may explain their low occurrence in the dataset.

Upon comparing the frequency of AA occurrence in our dataset with UniProtKB/Swiss-Prot (release 2023_01) (Fig. 6b), we noted an over-representation of K, R, T, and tryptophan (W), which is consistent with our earlier results. Interestingly, while W is infrequently present in UniProtKB/Swiss-Prot proteins (at a frequency of 1.1%), it is present in our dataset at a frequency exceeding 5%, indicating that it could play a significant role. Previous studies have demonstrated that the indole ring NH protons of W contribute to CEST contrast at approximately 5.5 ppm [80]. However, the CEST values in our dataset were measured at 3.6 ppm, suggesting that the amide group in the backbone, which resonates at this frequency, may be responsible for generating a signal at 3.6 ppm. The AAs that are underrepresented in our dataset are alanine (A), phenylalanine (F), isoleucine (I), and leucine (L), which are non-polar and hydrophobic, lacking amine or hydroxyl groups in their side chains, as well as glutamic acid (E) and aspartic acid (D), which are negatively charged. Because a peptide with high CEST contrast is required to be soluble in water, it is not surprising to find fewer hydrophobic AAs in the dataset.

Next, we conducted an analysis of the impact each AA may have during the evolutionary process (Fig. 6c). Using the ‘occurrence’ method described in the Materials and Methods section, we calculated the potential CEST value associated with each AA. Our results indicate that AAs with the highest associated CEST values are K, R, S, Q, I, and W, while T, F, Y, and the two negatively charged AAs, E and D, have relatively low CEST values. However, it should be noted that these values may vary depending on the context in which the AA is present, as CEST values are measured on a global peptide sequence. For instance, while W has a potential CEST value of approximately 20, the ’*KWR*’ motif has a CEST value of 17.27, and the peptides containing this motif have CEST values of 18.46 and 16.08. This initial analysis has allowed us to identify two groups of AAs. Specifically, we have observed that six AAs have a CEST value >15, which could potentially guide the evolutionary process towards the production of REs with significant weight. Conversely, the other AAs have a CEST value < 10.

Subsequently, we conducted a similar analysis on the 20 most prevalent motifs (ranging in size from 2 to 6) in the training set, as depicted in Fig. 6d. Since the focus of this study is on predicting peptides as short as 12 AAs, it is important to consider motifs that consist of only 2 AAs. As anticipated, motifs of size 2 and 3 dominate in the MDB. Notably, the most frequently occurring motifs consist of K or T. Although present, the divide between motifs with a CEST value greater than 10 and those below 10 is less noticeable. Many motifs with a high CEST contain K, R, and S, whereas motifs with low CEST values comprise T, E, and D. These findings are consistent with our earlier analyses and provide valuable insights for scrutinizing the performance of the evolutionary algorithm.

### Assigning random weights to REs in POET_*Regex*_

**Fig. 7:**
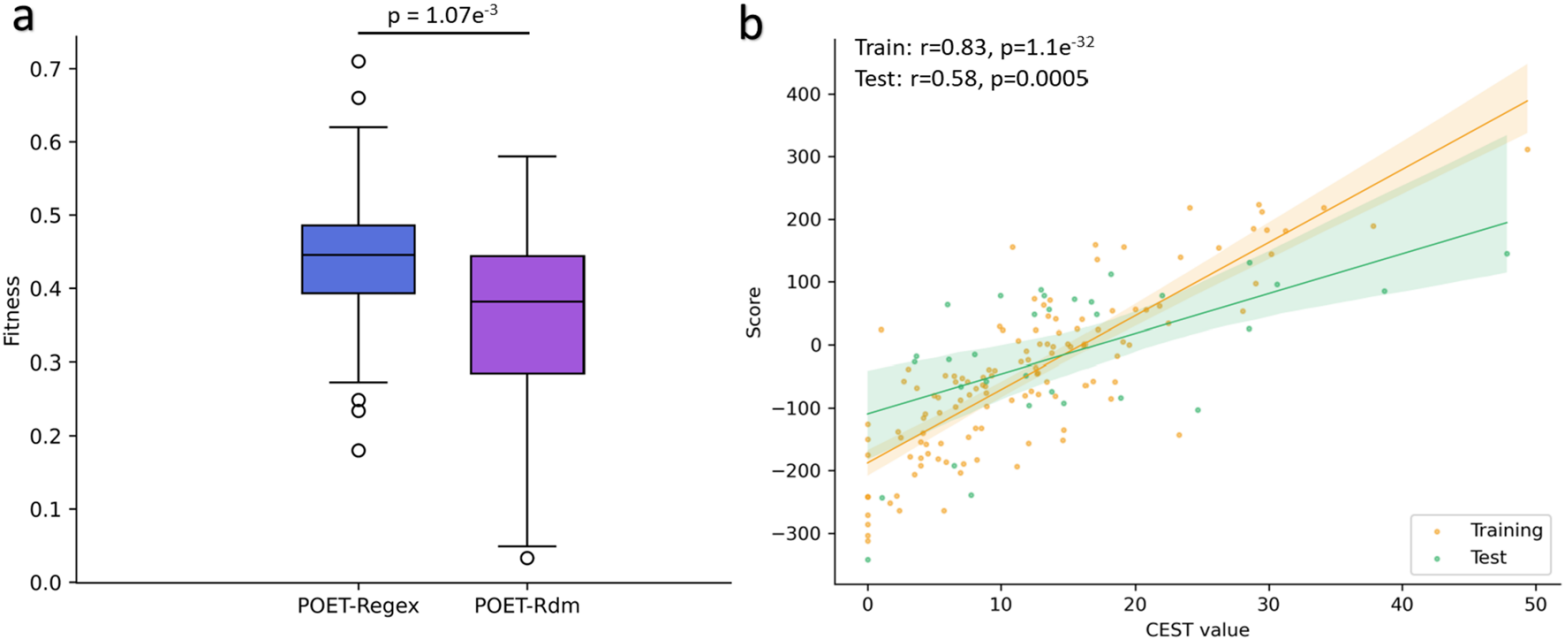
**a** Comparison of POET_*Regex*_ (blue) and POET_*Rdm*_ (purple) models on the test set. **b** Performance of the best POET_*Rdm*_ model on the training set (orange) and the test set (green). The translucent bands around the regression line represent the confidence interval for the regression estimate

To confirm the effectiveness of the training step (i.e., weight adjustment) during the evolutionary process, we conducted two independent experiments, each comprising 50 replicates, using identical parameters to those in Table 2. In the first experiment, POET_*Regex*_ was employed with weight adjustments (training step is active), whereas in the second, control experiment (called POET_*Rdm*_), weights are randomly defined during the initialization step and randomly changed with a probability of p=0.1 for each rule (training step is inactive). After selecting a rule for change, the mechanism of POET_*Rdm*_ replaces the rule’s weight with a new random value, uniformly sampled from the interval -10 and 10. The remaining parts of the two algorithms operate similarly. Choosing random weights in POET_*Rdm*_, as opposed to incorporating a training step, favors random exploration of weights over attempting to directly converge towards optimal weights. If random changes in weights result in an individual achieving higher fitness, there is a chance that tournament selection will choose this individual to contribute part of its genetic material to subsequent generations. The results of these experiments obtained on the test set are presented in Fig. 7a.

As expected, the results of the experiments with random weights (POET_*Rdm*_) are lower than the results of the experiments with the training step (POET_*Regex*_). A paired t-test was performed and confirmed that the difference is statistically significant (p-value=1.07e^−3^). Indeed, the average fitness value obtained on the test set with POET_*Rdm*_ is 0.359 compared to POET_*Regex*_ which is 0.443. The results of POET_*Regex*_ are about 23% (+0.084) higher than the experiments with POET_*Rdm*_. Among the POET_*Rdm*_ models, the best model (Fig. 7b) has a fitness value of 0.58 (with p-value=5.04e^-4^) on the test set. This fitness value is 0.13 ($\sim$22%) lower than the best model achieved using POET_*Regex*_. These results confirm the importance and efficiency of the training step during the execution of the algorithm.

### Best POET_*Regex*_, model obtained after the evolutionary process

**Fig. 8:**
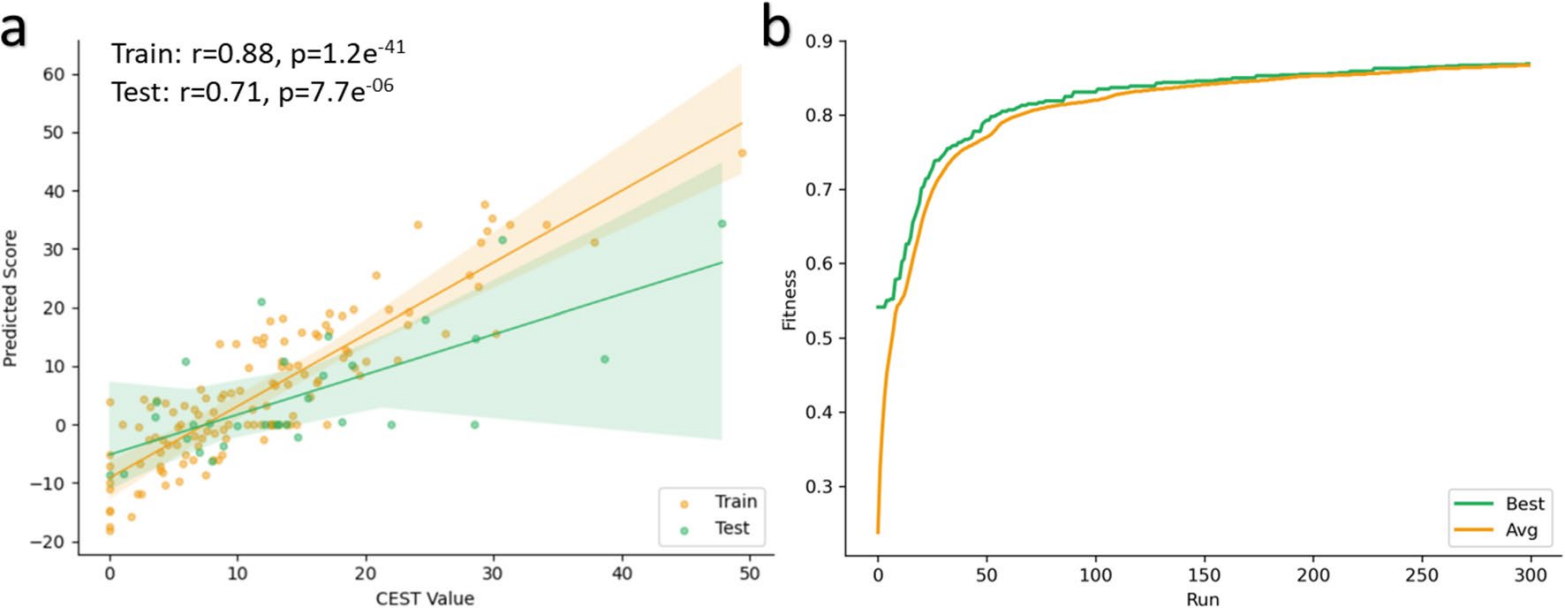
**a** Performance of the best POET_*Regex*_ model on the training set (orange) and on the test set (green). The strong correlation indicates that the algorithm has converged to a good solution. The translucent bands around the regression line represent the confidence interval for the regression estimate. **b** Evolution of the fitness value during the evolutionary process. The green curve represents the fitness value of the best individual, and the orange curve represents the fitness value of the entire population

Out of all the previous experiments, the best POET_*Regex*_ model (Additional file 2) exhibited interesting results with a strong correlation of 0.88 (p-value=1.2e^-41^) on the training set and 0.71 (p-value=7.7e^-6^) on the test set (Fig. 8a). A correlation exceeding 0.5 indicates a highly positive correlation between the predicted values of the model and the actual wet lab measurements. Furthermore, a p-value below 0.05 indicates that the results are statistically significant. As shown in Fig. 8b, the fitness values of the best individual and for the entire population continue to improve until around 100 generations and then tend to stabilize. This means that the algorithm converges to a good solution. It is interesting to note that this model comprises 29 rules, consisting of a combination of REs (80%) and contiguous motifs (20%). For instance, the ’*KL*’ motif is one of the contiguous motifs with a weight of 3.397. Finally, these results confirm that our GP algorithm is capable of evolving protein-function models adapted to the CEST problem. Consequently, the algorithm is effective in identifying motifs that can enhance the CEST signal.

### Comparison between POET_*Regex*_ and initial POET

In order to evaluate the efficiency of adding REs to build protein-function models, we conducted 100 experiments using the initial version of POET as a baseline for comparison. The initial version of POET has previously demonstrated effectiveness in predicting high CEST contrast peptides. In the initial algorithm, models consist of collections of evolved rules comprising sequences of peptide or AA patterns and a numerical weight indicating their importance in producing high contrasts. While these models are nonlinear, they employ a linear method to represent the discovered patterns in each rule. Our hypothesis is that REs can enhance motif discovery in POET_*Regex*_ and, in turn, increase the efficacy of the evolved models. For a fair comparison of the 2 programs, the same training set was used to train the POET and POET_*Regex*_ models, and the same test set was also used to evaluate them. The default parameters utilized in [53] were employed throughout the experiments.

**Fig. 9:**
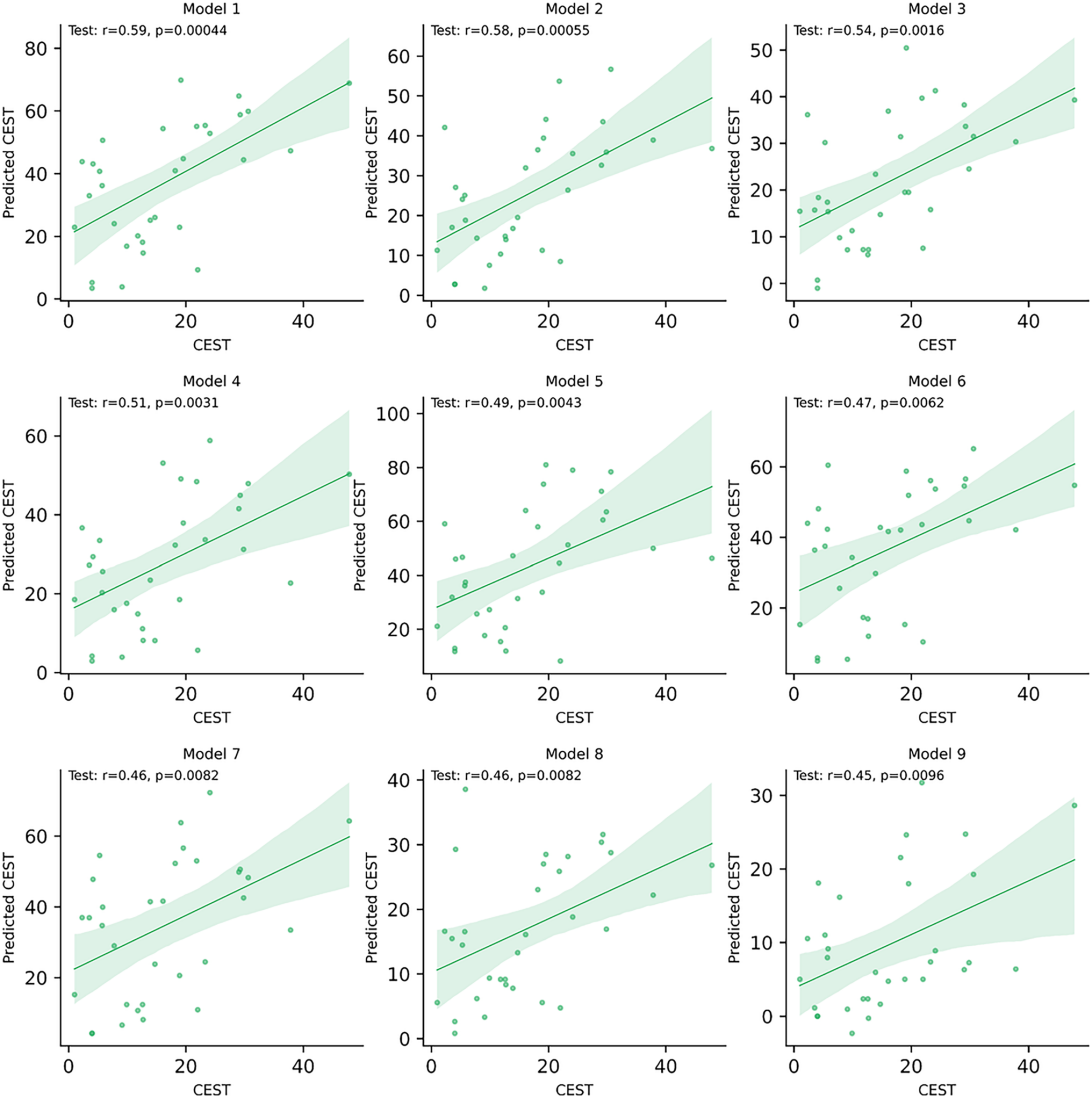
The 9 best POET models. Each dot represents a datapoint with a true CEST value associated with a predicted CEST value. The green line represents the regression line and the translucent bands around the regression line represent the confidence interval for the regression estimate

On average, POET exhibits a correlation of 0.292 and a p-value of 0.205. Some models drastically reduce the average because the evolutionary process was unable to find a good solution, or the algorithm converged too fast and got stuck in a local maximum. Therefore, we focus only on the 9 best models to take advantage of the best results. The average correlation of the top 9 POET models is 0.504 (average p-value of 4.68e^-3^), which is very close to the performance obtained by POET_*Regex*_. Fig. 9 displays the results of the top 9 POET models. Model 1 obtains the best performance with a correlation coefficient of 0.59 and a p-value of 4.4e^-4^, meaning the result is statistically significant. These results demonstrate the potential of the initial version of POET. However, the best POET_*Regex*_ model performed better than POET and indicates that REs add flexibility that POET does not have and improves the learning and prediction potential. The power and accuracy of the REs allowed the best POET_*Regex*_ model (among all replicates) to perform better with an increase in performance of 20% (+0.12).

### Peptide predictions with the best evolved model

After evolving the models and identifying the best one, we utilized the best model to predict peptides that could potentially outperform the gold-standard K12 peptides by exhibiting high CEST values. We employed a computational DE process in which the best POET_*Regex*_ model (Additional file 2) and the standard encoding with 20 AAs were used to predict new peptides. In this context, higher prediction scores correlate with higher CEST values. We conducted 3 experiments with varying numbers of cycles (1000, 100 and 10 cycles) during an *in silico* DE process. This approach replaces the DE screening step by selecting peptides with a potentially high CEST value using the best POET_*Regex*_ model and drastically reduces experimental time and costs. The results for peptides with the highest predicted score (top 1) and peptides with both a high predicted score and high hydrophilicity (best) for each experiment can be found in Table 3, while all predictions are available in Additional file 1: Table S3. It is important to highlight that in the DE process applied for peptide prediction, the higher the number of cycles, the more the peptides generated will be similar and converge towards an identical solution. Conversely, a limited number of cycles results in less accurate predictions, but it allows for broader exploration and the generation of original peptides. Thus, determining the optimal number of cycles is a key point in the employed DE.

**Table 3:** Predicted peptides with highest predicted score (Top 1) and best predicted peptides with highest hydrophilicity and high score (Best), with 1000, 100 and 10 cycles during DE

Cycles	Predicted peptide (Top 1)	Predicted score	Hydrophilicity
1000	ICKLLKLLKLLK	97.66	0.05
100	RLKSMQLKLDKL	82.83	3.25
10	QSCKYCQSLKFD	52.85	1.52
Cycles	Predicted peptide (Best)	Predicted score	Hydrophilicity
1000	QSLKQSIKKLKK	92.52	4.94
100	QDGSKKSLKSCK	74.55	5.37
10	SEVEKPFWEQDK	39.91	7.52

**Fig. 10:**
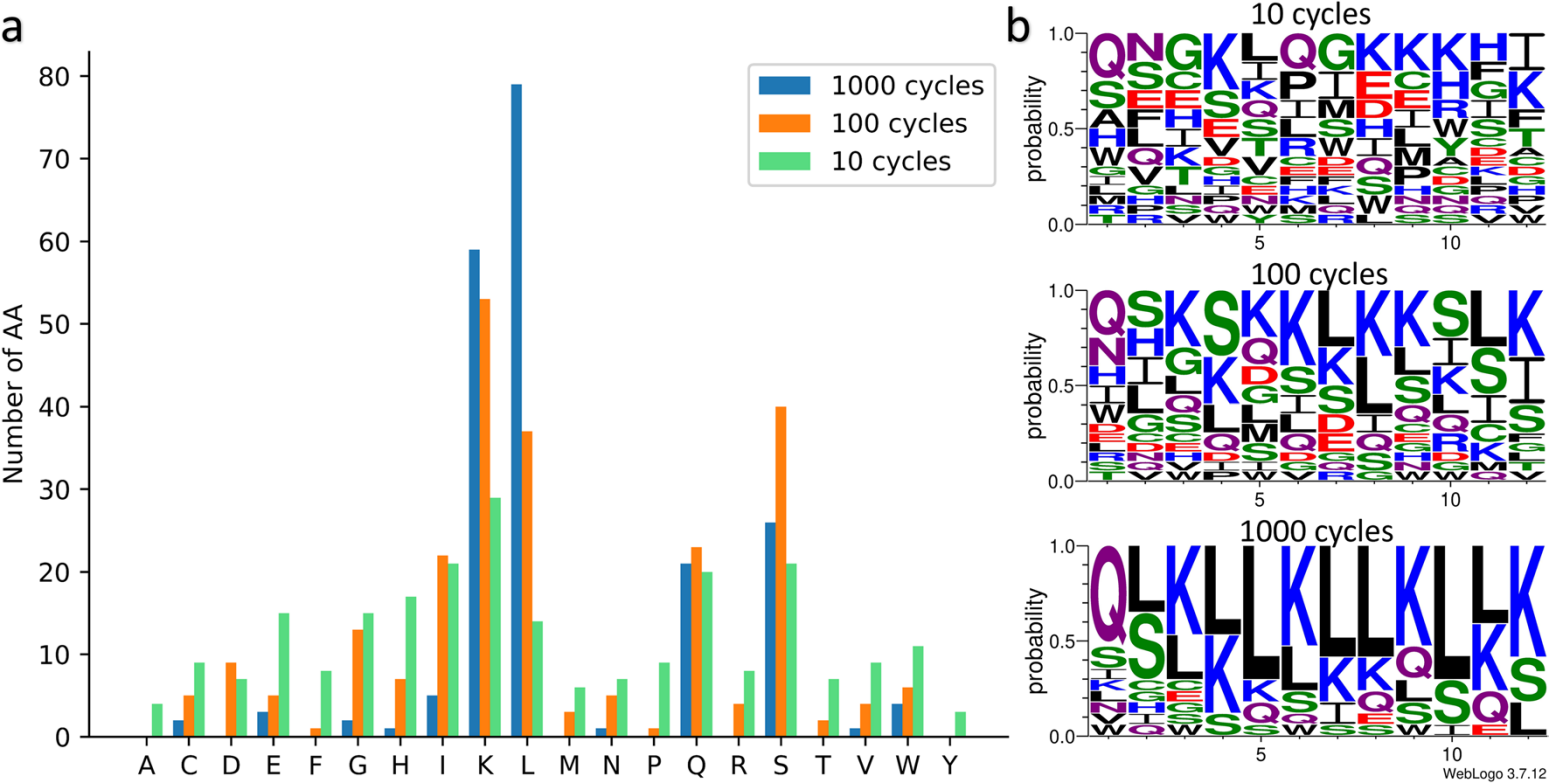
**a** Number of AAs present in the predicted peptides in the 3 types of DE experiments: 1000 (blue), 100 (orange) and 10 (green) cycles. **b** Sequence logos highlighting the probability of each AA at a given position, for the 3 experiments. As the number of cycles increases, the predicted peptides are more similar with high rates of lysine and leucine. The polar AAs are in green, the neutral in purple, the positively charged in blue, the negatively charged in red and the hydrophobic in black

Next, we analyze the AA composition of the predicted peptides. The results are illustrated in Fig. 10a. As expected, peptides generated after 1000 cycles exhibit a homogeneous AA composition achieving high predicted scores (>90). In contrast, peptides generated after 100 and 10 cycles display a more heterogeneous AA composition with lower scores (approximately 70-80 for 100 cycles and 40-50 for 10 cycles). The sequence logos in Fig. 10b generated with the WebLogo 3 tool [81], highlight the probability of each AA at a given position. With an increasing number of cycles, the presence of Q, L, S, and K becomes more prominent, confirming the tendency to converge towards similar peptides with a homogeneous AA composition.

Also, we observed a significant presence of isoleucine in predicted peptides in experiments involving 100 and 10 cycles (Additional file 1: Table S4). The abundance of lysine, glutamine, and serine in the predicted peptides is consistent with our initial analysis of the dataset. Lysine, a positively charged AA, plays a crucial role in detecting CEST signals. Glutamine and serine, non-charged polar AAs with amide and hydroxyl groups, respectively, facilitate proton exchange with water molecules. Hence, we expected to find these AAs in the predicted peptides. Conversely, we anticipated a high presence of arginine and tryptophan, given their abundance in the dataset. However, the peptides predicted for 10, 100, and 1000 cycles only contained 1.6%, 3.3%, and 0% arginine, respectively, and 4.5%, 2.5%, and 1.6% tryptophan. Interestingly, we observed a significant occurrence of leucine in the predicted peptides, with percentages of 5.83% for 10 cycles, 15.42% for 100 cycles, and 32.92% for 1000 cycles. This is notable because leucine is not very abundant in the dataset. Leucine, a hydrophobic AA, contradicts the preference for hydrophilic and soluble peptides in CEST experiments. However, leucine plays a key role in protein structure folding and has a strong tendency to form alpha helices while maintaining their stability. Consequently, we used the ColabFold tool [82] based on the AlphaFold2 model [34] to perform 3D structure predictions of the leucine-rich predicted peptides. The results presented in Additional file 1: Figure S1 demonstrate that the predicted patterns tend to form alpha helices. Thus, the model can identify leucine-rich motifs that play a significant role in the formation of specific secondary structures, such as the alpha helix. In this manner, the GP algorithm has produced original results. Despite our initial expectation of observing a substantial number of arginine, threonine, and tryptophan, it found and favored glutamine, leucine, and isoleucine. This suggests that the algorithm was capable of discovering motifs that contribute to the function and/or structure of the predicted peptides.

We identified the main motifs present in the predicted peptides for the three types of experiments. As anticipated, these motifs primarily consisted of the residues K, L, Q, S, and I. In the peptides predicted after 1000 cycles, the main motifs involve lysine and leucine, such as *LK* (45), *KL* (38), *LLK* (28), or *LKLL* (17). However, there are also motifs that incorporated other AAs, such as *LQS* (10) or *SLK* (16). In experiments involving fewer than 100 and 10 cycles, motifs such as *QS*, *GS*, *SI*, *SL*, and *SLK*, *LKS*, *IKK*, *LQS*, *QSL* were observed. These results confirm the ability of our algorithm to extract valuable information from the data and leverage it to generate peptides with potentially significant CEST values.

### Experimental validation of predicted peptides

The best protein-function model evolved by POET_*Regex*_ was used to generate novel peptides that have the potential to enhance CEST contrast. In order to validate the reliability of our approach, we selected the top 3 predicted peptides with higher hydrophilicity and high score from each DE experiment (10, 100, and 1000 cycles) and evaluated their performance in the wet lab.

**Fig. 11:**
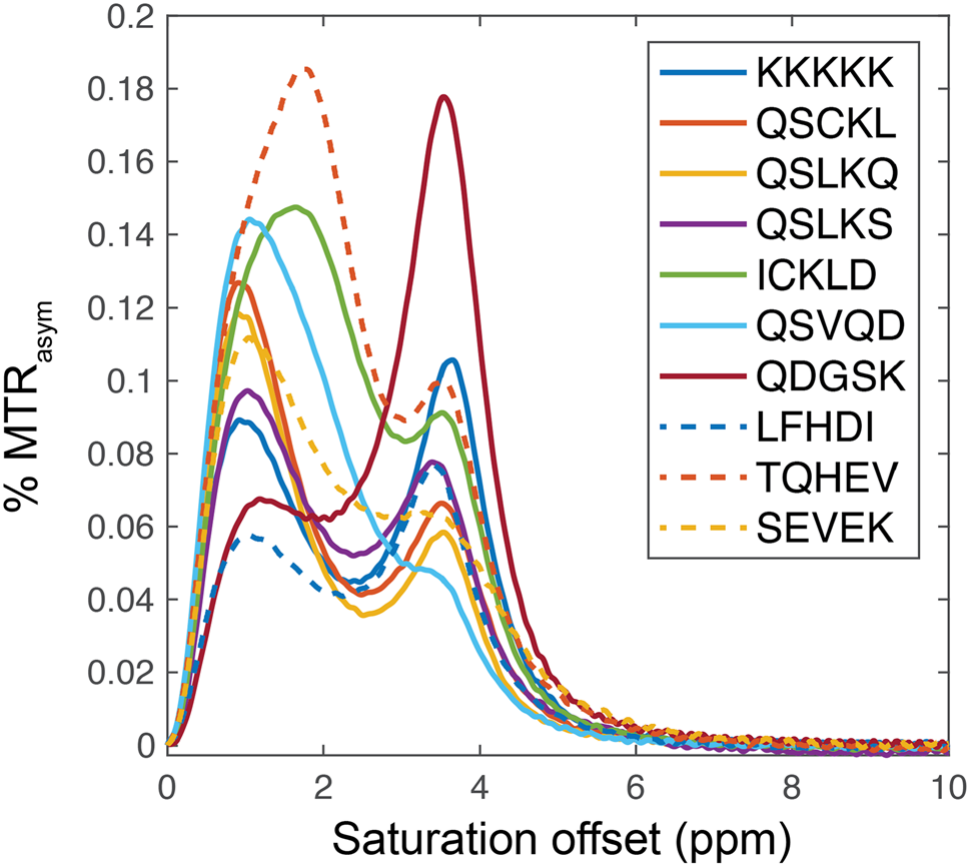
MTR_*asym*_ plot of nine peptides and the gold standard peptide (K12) measured by NMR

The 9 peptides were synthesized, and the magnetization transfer ratio asymmetry (MTR_*asym*_), a measure of CEST contrast, was obtained using NMR spectroscopy. The MTR_*asym*_ was normalized to the molar concentration of the peptide (Additional file 3: Table S1) and plotted as a function of the saturation frequency offset (Fig. 11). Since the POET_*Regex*_ was trained from the MTR_*asym*_ contrast at 3.6 ppm, the MRI results are presented in Table 4 for MTR_*asym*_ at 3.6 ppm. Data are normalized relative to the gold standard K12 peptide.

It is interesting to note that the results obtained from both the 1000-cycle and 10-cycle experiments do not demonstrate convincing results, showing an average MTR_*asym*_ of 6.47 (1000 cycles) and 7.67 (10 cycles). This outcome is likely due to either too many or too few cycles, leading to the generation of either too a too homogenous or a too diverse set of peptides. For instance, in the 1000-cycle experiments, 66% of the peptides consisted of QSLK or KLKK motifs, while no dominant motif was identified in the peptides from the 10-cycle experiments. These results highlight the limitations of our approach and allow us to explore relevant search spaces that are neither too constricted nor overly expansive, striking a balance between generating homogenous and overly diverse peptides. Conversely, among the 3 predicted peptides in the experiment with 100 cycles, peptide QDGSKKSLKSCK (QDGSK brown line in Fig. 11) generated MTR_*asym*_ 58% (17.59 MTR_*asym*_) larger than the gold standard peptide K12 at 3.6ppm (10.51 MTR_*asym*_). This prediction not only demonstrates superior CEST sensitivity, but also has a high predicted score and the highest hydrophilicity among the peptides considered (Table 3).

An interesting observation is that this peptide contains only 25% K residues, which is important for increasing the diversity of the AA composition of genetically encoded reporters [28]. QDGSKKSLKSCK is also unique compared to other peptides since it has a distinct peak at 3.6 ppm, resulting from the amide exchangeable protons, with little or no contribution from amine or guanidine exchangeable protons resonating between 1.8 and 2.0 ppm.

**Table 4:** Experimental results obtained in wet lab of the peptides predicted by POET_*Regex*_.

Peptides	# of cycles	POET_*Regex*_ score	MTR_asym (%)
KKKKKKKKKKKK	N.A	N.A	10.51
QSCKLKKLQSLK	1000	94.39	6.51
QSLKQSIKKLKK	1000	92.52	5.72
QSLKSWIEKLKK	1000	92.49	7.20
ICKLDKRIKKLK	100	80.52	8.96
QSVQDKLKKRII	100	77.18	4.36
**QDGSKKSLKSCK**	**100**	**74**.**55**	**17**.**59**
LFHDIEKQLKHA	10	43.79	7.01
TQHEVQSEKRGW	10	41.87	9.86
SEVEKPFWEQDK	10	39.91	6.14

Peptide depicted in bold has highest value of MTR\_asym.

These findings confirm that after training/evolving and employing the best POET_*Regex*_ model, the search space was successfully narrowed down, allowing us to highlight a candidate peptide that exhibits a performance exceeding 58% in comparison to the gold standard peptide K12. POET_*Regex*_ has proven its ability to extract motifs with compelling properties, facilitating the generation of peptides tailored to address specific problems.

## Discussion

### Challenges of computational approaches to peptide discovery

Peptides have emerged as highly promising candidates for therapeutic targets, biomarkers for disease diagnostics, and medical imaging [9], particularly as MRI CEST contrast agents [27]. They offer several advantages, including high specificity, biodegradability, minimal tissue accumulation, and low toxicity. However, they also present certain disadvantages, such as low oral bioavailability, limited membrane permeability, low solubility, and the expensive and time-consuming nature of their synthesis [83]. Due to the challenges associated with generating new peptides through traditional experimental methods, several computational approaches have emerged to aid in peptide discovery. Among these, ML and DL algorithms, including large language models (LLM) [84], have recently gained prominence and show significant potential in various fields, such as synthetic biology and protein engineering [85]. Indeed, numerous studies have explored the potential of these algorithms to design bioactive peptides and proteins. For example, Imai et al. [86] developed an approach based on three machine learning algorithms (Support Vector Machine, Random Forest, and Logistic Regression) to construct a predictive model and screen for bile acid-binding peptides. Repecka et al. [87] and Tucs et al. [88] trained generative adversarial networks to extract intrinsic relationships from natural proteins. Zhang et al. [89] used an LSTM model that generates peptides and combined it with a protein-peptide binding prediction model to screen a significant number of potentially active peptides. Dean et al. [90] developed a variational autoencoder to generate antimicrobial peptides. More recently, LLMs like protBERT [91] have been used for protein design and engineering. In this case, the pre-trained BERT model was fine-tuned to generate antihypertensive peptides. Another example is ProGen [92], a language models used to generate sequences of artificial proteins. However, the use of these algorithms presents challenges such as the complexity of models, difficulty interpreting results since DL models are often considered black boxes [93], and the high cost of training on GPU cards. Additionally, these models require large amounts of data. For instance, ProGen [92] used a training dataset consisting of 280 million proteins. In the realm of synthetic biology, the scarcity of experimental and curated data often remains a problem, as the available datasets are generally too small to make it practical to employ DL methods [94]. To overcome these limitations, alternative computational approaches, such as evolutionary algorithms, can be employed to identify candidate peptides more efficiently.

### GP approach for peptide discovery

The discovery of new peptides with potential for therapeutic or diagnostic purposes is a complex task that involves exploring a large search space. Unfortunately, exhaustive exploration of this space is not feasible with current methods. Indeed, this challenge is a NP-hard optimization problem, and the ratio between functional and non-functional proteins is heavily skewed toward non-functional ones. We have employed a heuristic approach based on Genetic Programming, which has proven effective in navigating complex search spaces where other methods may not perform as well. Inspired by evolutionary mechanisms, the GP algorithm is capable of finding satisfactory solutions to a given problem without prior knowledge, making it suitable for situations where solutions are not easily defined. GP allows to manipulate structures that perform actions (like programs), as opposed to other (evolutionary) optimization methods, which try to optimize a target function directly. The outcome of GP actions is what is optimized, but what is evolved is the structure which can change its complexity based on the demands of the problem. This decouples the structure from the behavior, a very important aspect of genetic programming [70, 95]. Here, we used REs as the structures to be evolved to identify motifs in peptide sequences. While REs are robust tools, the manual tuning of REs can be a time-consuming, tedious, and error-prone process [96]. Therefore, developing a method that can automatically generate REs and adapt their complexity for a given problem is a challenge but has the potential to significantly facilitate peptide discovery and protein engineering. In principle, GP can be further enhanced by using a multi-objective approach with a Pareto front of conflicting optimization goals, but we have chosen not complicate matters for this contribution.

### POET_*Regex*_ applied to the CEST problem

In this study, we introduce POET_*Regex*_, a new tool designed to evolve a protein-function model and discover new peptides for a given problem. To illustrate the feasibility of our method, we used POET_*Regex*_ to predict peptides with increased sensitivity detected by CEST. POET_*Regex*_ utilized GP to optimize protein-function models, represented by a list of REs. While the initial version of POET relied on a list of motifs of contiguous AAs, restricting peptide discovery, this new version incorporates two significant enhancements. First, it leverages the flexibility of RE to identify specific motifs, enabling a more expansive exploration of the search space. Second, the training step takes advantage of high-quality data generated in the laboratory to adjust RE weights, moving away from the random assignment approach. This step enhances the identification of motifs crucial to the specific problem. In addition, POET_*Regex*_ exhibits the ability to train on small datasets, distinguishing it from DL models. Previous studies have demonstrated the potential of combining algorithms with limited datasets to achieve interesting results [97, 98]. Finally, the use of RE ensures the complete transparency of the model. Indeed, while DL models are often regarded as black boxes that are challenging to interpret, our model, despite relying on initially complex RE, is fully explainable. This ensures comprehension during prediction, detection of biases, user confidence, and continuous model improvement, contributing to a more ethical and effective utilization of AI.

### The key points of POET_*Regex*_

By combining GP with REs, we achieved a 20% improvement in performance compared to the previous version of POET. While this combination proves to be an interesting and efficient solution, it’s important to acknowledge that motif search can be limited by the complexity of the motifs. Our approach relies on constructing a MDB that consists of a set of motifs found in the training dataset. Some motifs may be more complex and less prevalent, which can impact model training and subsequent predictions. Therefore, the construction of the MDB from the data is a key point of our study, and it’s likely that increasing the amount of data could improve the performance of our strategy.

Moreover, the ability of an RE to extract motifs is related to its length, which corresponds to the depth of the binary tree. To generate suitable REs, we adopted the ramped half-and-half method, which allows for the creation of a heterogeneous population of trees with varying depths. This approach strikes a balance between the complexity of RE and its ability to generalize to new data. However, using shallow trees can result in small RE that may lead to overfitting. These small REs can only extract specific motifs, limiting flexibility and hindering the ability of the model to generalize. Conversely, excessively deep trees produce long REs that may lead to underfitting. Long REs have the potential to extract a wide range of motifs, losing specificity, especially if there are numerous alternative choice operators (|). Additionally, large REs may contain regions that are not utilized during the training step but could play a significant role during the prediction step. These instances of "false positives" can introduce bias into the predictions.

Hence, it is crucial to select an appropriate RE size and number of REs to avoid overfitting, underfitting, and the propagation of non-exploited regions. Another key to the success of the model is its ability to generalize data, requiring that REs be heterogeneous, i.e., they do not extract the same motifs. It is worth noting that the best POET_*Regex*_ model (Additional file 2) primarily consists of variable-sized REs but also incorporates fixed-size motifs. These results, combined with the high correlation coefficient obtained during the training step, indicate that our algorithm can extract (from the data) essential and specific motifs to address the problem at hand while introducing the flexibility needed to generate innovative solutions.

Finally, it is important to note that POET_*Regex*_ can be trained multiple times to obtain better performance. By incorporating new experimental peptide data into the dataset and refining the hyperparameters of the model, we can enhance the performances of POET_*Regex*_, similar to the improvements observed in the initial POET version [53], where 8 epochs were realized.

### Peptide prediction with POET_*Regex*_

After the evolutionary process, we utilized the best POET_*Regex*_ model to generate new peptides with higher sensitivity of detection by CEST using a DE method, which is a powerful tool for protein engineering [30]. Traditional DE involves generating a population of individuals with similar characteristics to the desired outcome, but this approach often gets stuck in local optima due to the similarity of starting points. Additionally, it relies on performing mutations and wet lab evaluations (screening step), which can be time-consuming and expensive. By employing a model like POET_*Regex*_, we replace the screening step. The model can extract motifs of interest (or the inverse if the score is negative) to select the most promising peptides for the next generation. This broader coverage of the search space increases the likelihood of escaping from local optima. Furthermore, the extrapolation capacity of our model enables it to generate original peptide sequences. Indeed, the peptides designed by POET_*Regex*_ were found to be rich in lysine, serine, glutamine, leucine, and isoleucine, whereas the input data contained a high number of lysine, serine, threonine, and arginine and few glutamine, leucine, and isoleucine. This indicates that the model favored motifs with a higher frequency of amino acids lysine and leucine while avoiding motifs containing arginine and threonine. The significant presence of lysine is consistent due to its amine group and positive charge, but the inclusion of leucine is original as it is a hydrophobic AA. Predictions of the 3D structure of leucine-rich peptides suggest that this residue plays an important role in the three-dimensional conformation of the peptide. The generation of secondary structures contributes to the improved thermal stability of proteins [99]. In the future, by combining peptides obtained through the evolutionary algorithm with proteins, it may be possible to achieve a stable structure without compromising the potential for enhanced detection through CEST contrast. A similar approach has been successfully employed in the generation of *de novo* biosensors for CEST MRI by coupling proteins with peptides exhibiting high CEST potential [28]. Finally, nine peptides generated by POET_*Regex*_ were carefully selected, synthesized, and their MTR_*asym*_ values were calculated. Six of them (those with 10 and 1000 cycles) have enabled us to highlight the DE boundaries, while among the peptides with 100 cycles we have identified a potential candidate (QDGSKKSLKSCK) displaying a remarkable increase of over 58% compared to the gold standard K12. Although there was no apparent correlation between POET_*Regex*_ prediction scores and experimental outcomes (probably due to the limited number of peptides synthesized), this discrepancy might be attributed to the impact of either excessive or insufficient cycles during the DE. Nevertheless, we successfully identified and synthesized a promising candidate peptide that exhibited interesting characteristics when compared to genetically encoded reporters. These results demonstrate that our method is capable of evolving protein-function models to extract motifs that align with a given problem even in the presence of initial constraints. Moreover, it has the capacity to reduce the search space and leverage a more comprehensive range of amino acids. It is essential to emphasize that these results were obtained from a single epoch, implying that we can further improve the performance of the model and enhance the sensitivity of generated peptides by improving the dataset with additional experimental data.

## Conclusions

The development of the POET_*Regex*_ tool represents a significant advance in the field of protein engineering. This study highlights the effectiveness of combining genetic programming with regular expressions to efficiently explore a vast search space and generate new peptides, which could lead to the development of new therapeutic targets and biomarkers. Although our study focused on the use of POET_*Regex*_ to improve the sensitivity of CEST-based imaging, the program could also be applied to other areas of protein engineering. The flexibility of REs provides a precise, explainable, and targeted approach for identifying specific motifs, making POET_*Regex*_ applicable beyond the scope of our study. Considering the increasing prominence of personalized medicine and the expanding utilization of peptides in the pharmaceutical market, we firmly believe that *in silico* approaches like POET_*Regex*_ can play a crucial role in accelerating the discovery of new peptide targets. In this manuscript, our focus was on comparing POET_*Regex*_ with an initial version of the POET system. In both cases, a notable feature of POET has been its ability to be applied to small datasets. An ongoing effort in this research is to expand our comparison to include state-of-the-art algorithms. Our preliminary results indicate that the majority of such algorithms struggle to generate generalized models with small datasets. While using a linear sequence of AA alphabets has been a common and conventional method for representing protein sequences, other approaches such as Prot2Vec [100] have been proving fruitful for feature extraction from biological sequences. It is intriguing to observe how using a more sophisticated non-linear protein representation could enhance a system like POET.

## Supplementary Information

Below is the link to the electronic supplementary material.

Supplementary file 1 (docx 188 KB)

Supplementary file 2 (csv 2 KB)

Supplementary file 3 (xlsx 13 KB)

## Data Availability

The code and datasets analysed during the current study are available in the Gitlab repository, https://gitlab.com/NicolasScalzitti/poet_regex

## Abbreviations

POET: Protein optimization engineering tool
CEST: Chemical exchange saturation transfer
MRI: Magnetic resonance imaging
AA: Amino acid
DE: Directed evolution
ML: Machine learning
DL: Deep learning
AI: Artificial intelligence
GA: Genetic algorithm
GP: Genetic programming
RE: Regular expression
K12: Poly-lysine peptide
MDB: Motif database
EA: Evolutionary algorithm
MTR_*asym*_: Magnetization transfer ratio asymmetry
NMR: Nuclear magnetic resonance
LLM: Large language model
