# Supplementary material for: Computational peptide discovery with a genetic programming approach
Source: J Comput Aided Mol Des. 2024 Apr 3;38(1):17. doi: 10.1007/s10822-024-00558-0 (PMC11416381; doi:10.1007/s10822-024-00558-0)
Supplement: Supplementary file 1 — (docx 188 KB) [file 10822_2024_558_MOESM1_ESM.docx]

| **Sequence** | **CEST (3.6ppm)** | **Dataset** | **Sequence** | **CEST (3.6ppm)** | **Dataset** |
| --- | --- | --- | --- | --- | --- |
| LWSDIKMKLKKT | 49.37196 | Train | IRRWNDRIRITS | 13.90809 | Train |
| AKCKVQSANVCK | 37.82153 | Train | TTTKTTTKTTTK | 13.8 | Train |
| KMWDWEQKKKWI | 34.11149 | Train | PIKQIAWPIIEH | 13.6599 | Train |
| WDWEQKKKWI | 31.26163 | Train | KVLWRMPAQIIQ | 13.51793 | Train |
| VTRMTIQVKGSK | 30.16713 | Train | WDRTSTRPSSVL | 13.46 | Train |
| TSKSKKRMTAKK | 29.83349 | Train | KEEVWLKWLI | 13.42497 | Train |
| ICLKSQPICGID | 29.49547 | Train | KSSKSSKSSKSS | 13.2 | Train |
| LELKLGKRPMGW | 29.24477 | Train | IIRSPICCVSRV | 12.89188 | Train |
| ARNRKKIMMRWI | 29.01464 | Train | RSRSRSRSRSRS | 12.8 | Train |
| PVNRLGKMSKNR | 28.83256 | Train | GRKRGAIWKDTK | 12.75 | Train |
| NVVVQRRNHHTS | 28.05341 | Train | KHKHKHKHKHKH | 12.7 | Train |
| VGSVKSGNLRMR | 26.22986 | Train | LSQQPRKRATWR | 12.60838 | Train |
| KMWDWEQKKKWI | 24.09143 | Train | TTKTTKTTKTTK | 12.6 | Train |
| QCAGWVQKRQIQ | 23.37685 | Train | DKVCKIQKRKWH | 12.51172 | Train |
| KGKLDKDRNL | 23.28052 | Train | KKKKKKKKKKKK | 12.5 | Train |
| KYTKTRKQSSKA | 22.48 | Train | RLWNSGEGRGEN | 12.26758 | Train |
| VCNRIEPLKPIL | 21.82239 | Train | QCRAGAMPAMYV | 12.05809 | Train |
| ERQEEKIKKW | 20.79427 | Train | KKRLHWIRWHCG | 12.01536 | Train |
| RKHHGWRWEQWK | 20.00466 | Train | KKRKKHKKGKKP | 11.9 | Train |
| CQLAWRPCAKAS | 19.532 | Train | KGGKGGKGGKGG | 11.8 | Train |
| DKRKIKQKMWWG | 19.13841 | Train | LDHTWGKWGHQS | 11.50256 | Train |
| WFGLQRHLKKKD | 19.0715 | Train | KKGKKKGKKHKK | 11.3 | Train |
| `PVARKVVQICHP | 18.63698 | Train | PGGVRSNDLLEV | 11.18031 | Train |
| CCWHNPKWRRTR | 18.46 | Train | DKRKIKQKMWWG | 10.86 | Train |
| PRSWEVKEKETM | 18.27337 | Train | KGKGKGKGKGKG | 10.8 | Train |
| LHSSQWLKVDHLL | 18.17873 | Train | CHLKDLRKMGLR | 10.1388 | Train |
| RGRGRGRGRGRG | 17.2 | Train | KKAKKKGKKHKK | 9.9 | Train |
| RGKMPLRWMTRK | 17.14 | Train | RRCQAQEFWLGA | 9.515134 | Train |
| KSKSKSKSKSKS | 17 | Train | KKGKKKGKKPKK | 9.3 | Train |
| SSDQDRDKWL | 16.82505 | Train | DSSSSSDSSSSS | 9.1 | Train |
| RTTRTTRTTRTT | 16.3 | Train | KVIRYVVAPMKL | 8.94 | Train |
| MWQMKWTRKTRE | 16.24 | Train | NAPWKHWRIINE | 8.898582 | Train |
| KPWHGCASRTKR | 16.19014 | Train | NDISMCNKNNNW | 8.824446 | Train |
| HIKWRLTKGTRT | 16.08 | Train | KDRTSKPKRPWC | 8.67 | Train |
| VSLQCWELGPNK | 15.70919 | Train | SDGSKIKDRD | 8.630329 | Train |
| KRIIEDDQLE | 15.19034 | Train | VINKVISNPCVN | 8.516833 | Train |
| RRMVNRTITRMW | 15.01 | Train | KVRCLVEARPSW | 8.194995 | Train |
| GNKKNWRWYKNR | 14.71334 | Train | VINKVISCPCVN | 8.109024 | Train |
| KWVVRPRIRRLL | 14.62156 | Train | VINKVISNPCVN | 8.107188 | Train |
| VSVVATGCVWET | 14.33556 | Train | GIFKTTKCKHNS | 7.61 | Train |
| TKTKTKTKTKTK | 14.1 | Train | QRHDSHRHGLWL | 7.543669 | Train |
| NSSNHSNNMPCQ | 14.06 | Train | HDDKNKESDD | 7.479407 | Train |
| DTTTTTDTTTTT | 7.2 | Train | KMGKLIGIPVLK | 47.83688 | Test |
| LSNRRGREQYAG | 7.08 | Train | MKVAAAMAPKQV | 38.66109 | Test |
| MAMADAAAPMNA | 6.965346 | Train | RLPKRVQGNVEK | 30.61573 | Test |
| NQYSNWNKNYK | 6.94 | Train | IGVLRSVKQTVR | 28.55074 | Test |
| EMRQWKWMWENA | 6.580628 | Train | NKQRRMLSRERS | 28.52089 | Test |
| WWWKPKREDFMK | 6.58 | Train | EPSNLPKGMNEK | 24.69024 | Test |
| RPPMLNVVRVVG | 6.489129 | Train | RRRRRRRRRRRR | 22 | Test |
| ETTTTTETTTTT | 5.9 | Train | RTTTRTTTRTTT | 18.9 | Test |
| GPMPMNAKMKLC | 5.81 | Train | NFLRAQRQCQKQ | 18.16368 | Test |
| ETNVRVKVVSES | 5.707696 | Train | HWSTCTRTRTLS | 17.1 | Test |
| RHRHRHRHRHRH | 5.5 | Train | KPCKWAGRACAK | 16.69529 | Test |
| NNKCQVVAAFVM | 5.401218 | Train | HGRKWKRTKFDD | 15.49 | Test |
| HLVVSPRVSWGC | 5.30282 | Train | AQCCQHRKGYMN | 14.69376 | Test |
| FNSNKITPTSNM | 5.29 | Train | TTTTTKTTTTTK | 13.8 | Test |
| VPNIQVKGSK | 4.99326 | Train | RKHHGWRWEQWK | 13.59362 | Test |
| VNLPMVMPNLRM | 4.53 | Train | MWVKGMKHKKMK | 13.23495 | Test |
| ETTETTETTETT | 4.4 | Train | KSSSKSSSKSSS | 13 | Test |
| PVVYKTVIQCCD | 4.333159 | Train | KGGGKGGGKGGG | 12.1 | Test |
| VAWVMKAHVCTM | 4.201038 | Train | GQRWLYKMKDSM | 11.86265 | Test |
| VNSDPSNGQMRD | 4.15 | Train | IKGMNIKMPTDQ | 9.95 | Test |
| DTTDTTDTTDTT | 4 | Train | GNCPMKVCSPMG | 8.89 | Test |
| DTTTDTTTDTTT | 4 | Train | IRTYLRKRNSTQ | 8.03 | Test |
| ETTTETTTETTT | 4 | Train | TVSEPVMMVSVS | 7.771304 | Test |
| NRVTESVRNVKM | 3.683273 | Train | DSSSDSSSDSSS | 7 | Test |
| GLGNQHVVVLGV | 3.528919 | Train | TTTTTTTTTTTT | 6.5 | Test |
| NWRDCLSLIVPN | 3.179373 | Train | GGRVWEWNVAA | 6.08351 | Test |
| LLRLLGLVER | 3.037419 | Train | SNHKMSECRGLR | 5.98 | Test |
| GLIEARAMQQCC | 2.704976 | Train | QTATENSQMNSG | 3.64 | Test |
| DSDSDSDSDSDS | 2.5 | Train | MAALLYQHRLARR | 3.528221 | Test |
| ETNVRVKVVSES | 2.368102 | Train | QTEHYENSARNS | 1.09 | Test |
| QERRDDILWD | 2.296864 | Train | DWNNYLYQNLH | 0 | Test |
| DTDTDTDTDTDT | 2.2 | Train |  |  |  |
| ETETETETETET | 1.7 | Train |  |  |  |
| RTRTRTRTRTRT | 1 | Train |  |  |  |
| NWNWWGLSYLA | 0 | Train |  |  |  |
| NGTLYLNNYYE | 0 | Train |  |  |  |
| NCGVNLVNAVGQ | 0 | Train |  |  |  |
| CNNIQGRNNSVW | 0 | Train |  |  |  |
| HIAVVNWVNVGH | 0 | Train |  |  |  |
| VLTWSAVNNNVQ | 0 | Train |  |  |  |
| NENQWHYYWRQ | 0 | Train |  |  |  |
| ELNTGLVLVNWK | 0 | Train |  |  |  |
| SYYWLWWHQQI | 0 | Train |  |  |  |

Table S1 – All data composing training and test sets used in the study.

| **Amino Acid** | **Hydrophobicity** | **Amino Acid** | **Hydrophobicity** |
| --- | --- | --- | --- |
| A | 0.17 | M | -0.23 |
| C | -0.24 | N | 0.42 |
| D | 1.23 | P | 0.45 |
| E | 2.02 | Q | 0.58 |
| F | -1.13 | R | 0.81 |
| G | 0.01 | S | 0.13 |
| H | 0.96 | T | 0.14 |
| I | -0.31 | V | 0.07 |
| K | 0.99 | W | -1.85 |
| L | -0.56 | Y | -0.94 |

Table S2 – Hydrophobicity of each amino acid according to **Rose et *al,.*** [1]. Blue cells represent hydrophobic amino acids.

| **Peptide** | **Hydrophilicity** | **Score** | **Cycle** |
| --- | --- | --- | --- |
| ICKLLKLLKLLK | 0.05 | 97.66 | 1000 |
| QSLKLLKLQSLK | 1.59 | 95.63 | 1000 |
| WIEKLLKLQSLK | 1.3 | 94.7 | 1000 |
| QSCKLKKLQSLK | 3.46 | 94.39 | 1000 |
| VQSLKLLKLLKL | 0.39 | 93.99 | 1000 |
| VQSLKLLKLLKL | 0.39 | 93.99 | 1000 |
| QSGSLKLLKLLK | 1.02 | 93.88 | 1000 |
| QSGSLKLLKLLK | 1.02 | 93.88 | 1000 |
| KLKKLQSLKLLK | 2.86 | 93.84 | 1000 |
| QLKLLKLQSLKL | 0.9 | 93.84 | 1000 |
| QLKLQSLKLLKL | 0.9 | 93.84 | 1000 |
| SLKLLKLLKLQS | 0.45 | 93.84 | 1000 |
| QLKLLKLLKLQS | 0.9 | 93.84 | 1000 |
| NHWSLKLLKSLK | 0.52 | 93.03 | 1000 |
| LLKLLKLQSLKS | 0.45 | 93.03 | 1000 |
| SGKLKKLLKLQS | 2.57 | 92.6 | 1000 |
| **QSLKQSIKKLKK** | 4.94 | 92.52 | 1000 |
| QSLKSWIEKLKK | 3.54 | 92.49 | 1000 |
| QSLKLLKSWIEK | 1.99 | 92.49 | 1000 |
| QSLKSWIEKLKK | 3.54 | 92.49 | 1000 |
| RLKSMQLKLDKL | 3.25 | 82.83 | 100 |
| ICKLDKRIKKLK | 5.01 | 80.52 | 100 |
| HIKKLKKLKWII | 2.01 | 79.34 | 100 |
| WIEKQDKQSLKS | 4.92 | 79.21 | 100 |
| QSVQDKLKKRII | 5.19 | 77.18 | 100 |
| ELKSQLKQGSLK | 4.74 | 76.98 | 100 |
| NHGSWIEKNRLK | 4.03 | 76.58 | 100 |
| SLQSLKDLQSCK | 2.84 | 76.33 | 100 |
| NVQLKLDKLQSG | 3.32 | 74.92 | 100 |
| **QDGSKKSLKSCK** | 5.37 | 74.55 | 100 |
| NHCPKSLKSILK | 3.39 | 74.35 | 100 |
| IGKDGSLKSGSI | 2.45 | 74.24 | 100 |
| DGSIKKGSLKSV | 3.81 | 73.71 | 100 |
| QHWSMQSLKSSI | 0.68 | 73.22 | 100 |
| WIKKSIEKWIIS | 0.31 | 72.75 | 100 |
| HSGSIKKLQSMT | 2.96 | 72.02 | 100 |
| QNHQGKLLKLQS | 3.56 | 71.64 | 100 |
| TSLKSGSGCILK | 0.86 | 71.52 | 100 |
| LQSLKVQSEKLF | 2.68 | 71.5 | 100 |
| QSLKQSLIHQSI | 2.34 | 71.44 | 100 |
| QSCKYCQSLKFD | 1.52 | 52.58 | 10 |
| APCPKQGKIGII | 2.48 | 49.88 | 10 |
| IVTSNRGSKKLK | 3.81 | 49.46 | 10 |
| WQGKTRGKPARI | 2.8 | 48.57 | 10 |
| WLEVQLIWERGK | 1.37 | 45.24 | 10 |
| QNLKLPMQHWSV | 0.98 | 44.86 | 10 |
| QLKGSQSIPYVT | 1.27 | 44.84 | 10 |
| LFHDIEKQLKHA | 5.34 | 43.79 | 10 |
| QHIWIPIEKWET | 2.53 | 41.98 | 10 |
| QSNHWSELIKPI | 2.65 | 41.95 | 10 |
| TQHEVQSEKRGW | 6.46 | 41.87 | 10 |
| SETQVQLKMCCK | 4.23 | 41.54 | 10 |
| RFIIQHWDCNHG | 1.13 | 40.97 | 10 |
| AREKLIMHQSFP | 3.88 | 40.94 | 10 |
| GVKKLIGHCHHC | 3.6 | 40.17 | 10 |
| **SEVEKPFWEQDK** | 7.52 | 39.91 | 10 |
| SNGSCKWIMDIH | 0.93 | 39.45 | 10 |
| HGSVTMIESYKF | 1.84 | 39.02 | 10 |
| HSGKSLDDNHQF | 4.95 | 39.01 | 10 |
| MNGSEFRKKHSI | 4.79 | 38.78 | 10 |

Table S3 – All peptides predicted by POET_Regex_ with 1000, 100 and 10 cycles.

|  | Cycles | | | | | |
| --- | --- | --- | --- | --- | --- | --- |
| AA | 10 | | 100 | | 1000 | |
| K | 29 | 12.08% | 53 | 22.08% | 59 | 24.58% |
| L | 14 | 5.83% | 37 | 15.42% | 79 | 32.92% |
| Q | 20 | 8.33% | 23 | 9.58% | 21 | 8.75% |
| S | 21 | 8.75% | 40 | 16.67% | 26 | 10.83% |
| I | 21 | 8.75% | 22 | 9.17% | 5 | 2.08% |

Table S4 – Occurrence of 5 AAs (Lysine, Leucine, Glutamine, Serine and Isoleucine) in the predicted peptides for each experiment (cycles 1000, 100, 10).


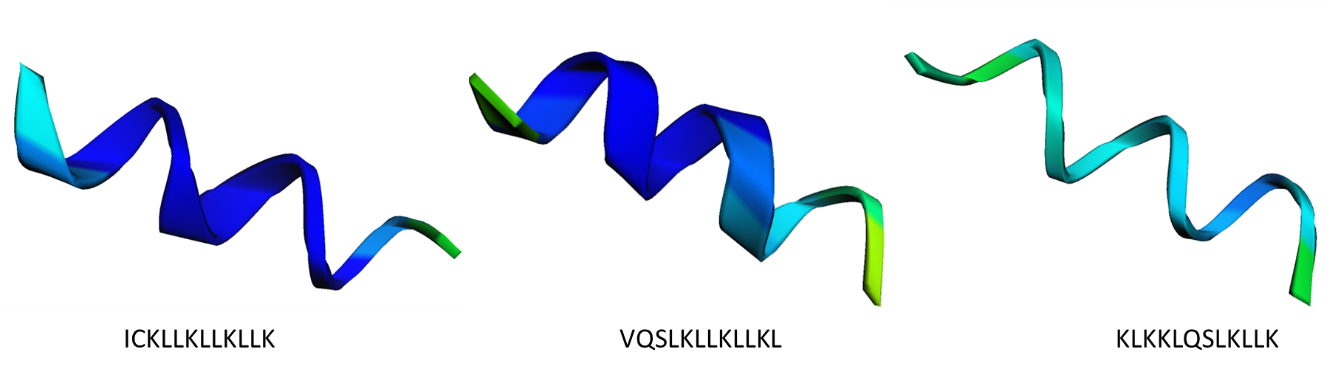


**Figure S1 –** Tridimensional structure of lysine rich predicted peptides.

**REFERENCES**

[1] George D. Rose, Ari R. Geselowitz, Glenn J. Lesser, Richard H. Lee and Micheal H. Zehfus. Hydrophobicity of amino acid residues in globular proteins. *Science,* 229(4716): 834-838, 1985
